# Fusion protein-driven IGF-IR/PI3K/AKT signals deregulate Hippo pathway promoting oncogenic cooperation of YAP1 and FUS-DDIT3 in myxoid liposarcoma

**DOI:** 10.1038/s41389-022-00394-7

**Published:** 2022-04-22

**Authors:** Ruth Berthold, Ilka Isfort, Cihan Erkut, Lorena Heinst, Inga Grünewald, Eva Wardelmann, Thomas Kindler, Pierre Åman, Thomas G. P. Grünewald, Florencia Cidre-Aranaz, Marcel Trautmann, Stefan Fröhling, Claudia Scholl, Wolfgang Hartmann

**Affiliations:** 1 Division of Translational Pathology, Gerhard-Domagk-Institute of Pathology, Münster University Hospital, Münster, Germany; 2 Gerhard-Domagk-Institute of Pathology, Münster University Hospital, Münster, Germany; 3 Division of Applied Functional Genomics, German Cancer Research Center (DKFZ) and National Center for Tumor Diseases (NCT), Heidelberg, Germany; 4 University Cancer Center, University Medical Center of Mainz, Mainz, Germany; 5 German Cancer Consortium (DKTK), Mainz, Germany; 6 Department of Laboratory Medicine, Institute of Biomedicine, Sahlgrenska Center for Cancer Research, Sahlgrenska Academy at University of Gothenburg, Gothenburg, Sweden; 7 Division of Translational Pediatric Sarcoma Research, German Cancer Research Center (DKFZ), Heidelberg, Germany; 8 Hopp Children’s Cancer Center (KiTZ), Heidelberg, Germany; 9 Institute of Pathology, Heidelberg University Hospital, Heidelberg, Germany; 10 German Cancer Consortium (DKTK), Heidelberg, Germany; 11 Division of Translational Medical Oncology, National Center for Tumor Diseases (NCT) and German Cancer Research Center (DKFZ), Heidelberg, Germany

**Keywords:** Sarcoma, Oncogenes, Growth factor signalling, Molecular biology

## Abstract

Myxoid liposarcoma (MLS) represents a common subtype of liposarcoma molecularly characterized by a recurrent chromosomal translocation that generates a chimeric *FUS-DDIT3* fusion gene. The FUS-DDIT3 oncoprotein has been shown to be crucial in MLS pathogenesis. Acting as a transcriptional dysregulator, FUS-DDIT3 stimulates proliferation and interferes with adipogenic differentiation. As the fusion protein represents a therapeutically challenging target, a profound understanding of MLS biology is elementary to uncover FUS-DDIT3-dependent molecular vulnerabilities. Recently, a specific reliance on the Hippo pathway effector and transcriptional co-regulator YAP1 was detected in MLS; however, details on the molecular mechanism of FUS-DDIT3-dependent YAP1 activation, and YAP1´s precise mode of action remain unclear. In elaborate in vitro studies, employing RNA interference-based approaches, small-molecule inhibitors, and stimulation experiments with IGF-II, we show that FUS-DDIT3-driven IGF-IR/PI3K/AKT signaling promotes stability and nuclear accumulation of YAP1 via deregulation of the Hippo pathway. Co-immunoprecipitation and proximity ligation assays revealed nuclear co-localization of FUS-DDIT3 and YAP1/TEAD in FUS-DDIT3-expressing mesenchymal stem cells and MLS cell lines. Transcriptome sequencing of MLS cells demonstrated that FUS-DDIT3 and YAP1 co-regulate oncogenic gene signatures related to proliferation, cell cycle progression, apoptosis, and adipogenesis. In adipogenic differentiation assays, we show that YAP1 critically contributes to FUS-DDIT3-mediated adipogenic differentiation arrest. Taken together, our study provides mechanistic insights into a complex FUS-DDIT3-driven network involving IGF-IR/PI3K/AKT signals acting on Hippo/YAP1, and uncovers substantial cooperative effects of YAP1 and FUS-DDIT3 in the pathogenesis of MLS.

## Introduction

Myxoid liposarcoma (MLS) accounts for 20–30% of malignant adipocytic tumors and about 5% of soft tissue sarcomas. It typically arises in the deep soft tissue of the extremities and represents the most frequent liposarcoma subtype in patients below the age of 20 years. Clinically, MLS is characterized by a high rate of local recurrence and development of distant, often extrapulmonary, metastases in ~40% of patients [1, 2]. Current therapeutic approaches are based on conventional strategies, including wide surgical excision, radiotherapy, and/or cytotoxic regimens based on anthracyclines and ifosfamide, recently complemented with newer agents such as trabectedin and eribulin [3, 4].

Genetically, most MLS are characterized by a t(12;16)(q13;p11) chromosomal translocation, which joins the *FUS* and *DDIT3* genes [5, 6]. The resulting chimeric FUS-DDIT3 protein acts as a transcriptional dysregulator that is essential for MLS pathogenesis, partially through interference with adipogenic differentiation [7–9]. Although FUS-DDIT3 is regarded as the predominant MLS driver, its exact mode of action, including its connection with FUS-DDIT3-dependent and other signaling inputs, remains incompletely understood. FUS-DDIT3 comprises the N-terminal, non-enzymatic portion of FUS and full-length DDIT3, a transcription factor harboring a DNA-binding domain [5]. In contrast to fusion proteins with enzymatic activity, e.g. BCR-ABL1 in chronic myelogenous leukemia [10], pharmacologic inhibition of the chimeric transcription factor FUS-DDIT3 represents a challenge due to the lack of defined small-molecule-binding pockets. Therefore, current research efforts focus on identifying downstream effectors deregulated by FUS-DDIT3 and proteins that act in concert with the fusion protein to modulate its oncogenic function indirectly.

Previous studies revealed that MLS cells depend on enhanced IGF-IR/PI3K/AKT signaling mediated through *PIK3CA* or *PTEN* alterations or, primarily, aberrant signal transduction networks governed by FUS-DDIT3, which was shown to drive *IGF2* expression, resulting in an IGF-II/IGF-IR transactivation loop [11–14]. Accordingly, recent preclinical studies demonstrated a molecular rationale for IGF-IR- or PI3K-targeted therapeutic approaches in MLS [11, 14]. In addition, the transcriptional co-activator YAP1 was uncovered as an essential MLS oncoprotein whose expression and nuclear activity are regulated by FUS-DDIT3; however, the mechanism of YAP1 activation in MLS, including its nuclear shuttling, is incompletely understood [15]. YAP1 plays an integral role in regulating cell proliferation and survival, and the restriction of its activity by the Hippo signaling pathway is critical for limiting tissue growth and organ size [16, 17]. Specifically, phosphorylation by the tumor suppressor kinases LATS1/2, in complex with the regulatory subunit MOB1, leads to YAP1 nuclear exclusion and/or proteasomal degradation. Upon inactivation of upstream Hippo kinases, YAP1 translocates into the nucleus where it associates with transcription factors, such as TEAD, to regulate target gene expression [16]. Pharmacologic inhibition of YAP1 activity with verteporfin was shown to suppress MLS cell viability in vitro and in vivo, rendering YAP1 a molecular target for therapeutic intervention in MLS [15, 18].

In this study, we aimed to elucidate the molecular mechanism of FUS-DDIT3-dependent YAP1 activation and to gain deeper insights into YAP1’s specific mode of action in MLS.

## Results

### FUS-DDIT3 promotes concurrent activation of IGF-IR/PI3K/AKT signaling and nuclear localization of YAP1

**Fig. 1:**
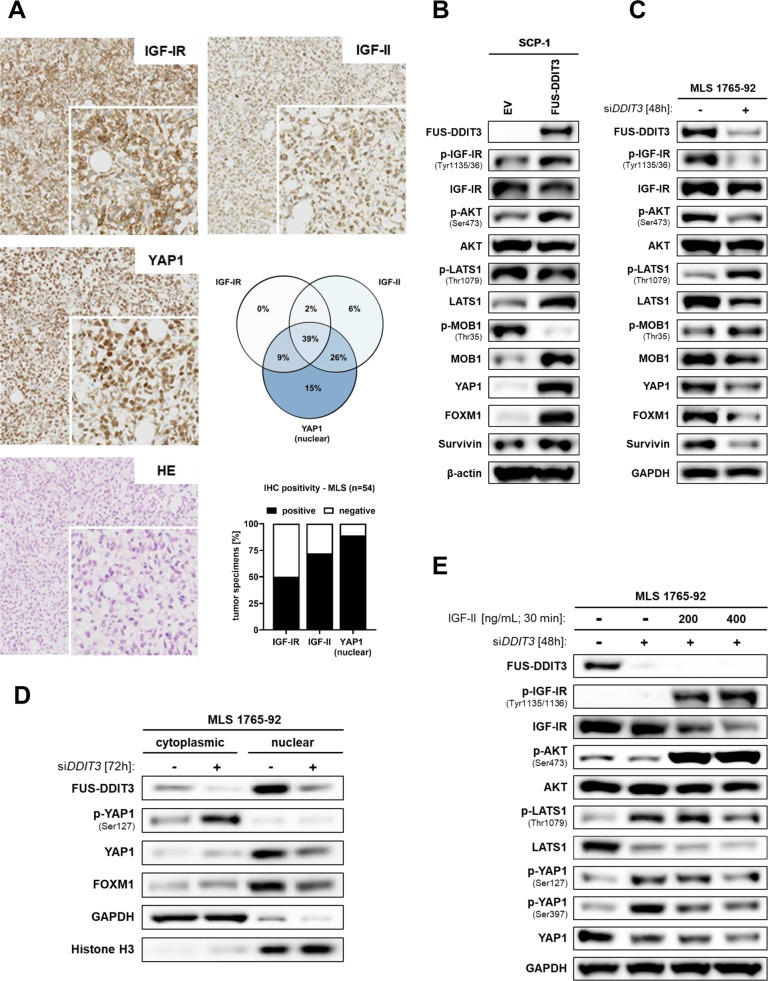
Concomitant activation of IGF-IR/PI3K/AKT signaling and YAP1 in MLS tumor specimens and in vitro models. **A** IHC stainings show strong expression of IGF-IR, IGF-II, and nuclear YAP1 in a representative MLS tissue specimen (original magnification, ×10; inset ×20). Venn diagram representing the overall concordance of IHC positivity (%). Bar chart summarizing overall IHC positivity of IGF-IR, IGF-II, and YAP1 of the MLS cohort (*n* = 54). **B** Immunoblots of SCP-1 mesenchymal stem cells transduced with FUS-DDIT3 or EV. **C** Effects of *FUS-DDIT3* depletion on the IGF-IR/PI3K/AKT and Hippo/YAP1 pathways in MLS 1765-92 cells. **D** Subcellular fractionation of MLS 1765-92 cell lysates showing increased cytoplasmic p-YAP1 (Ser127) and diminished total YAP1 levels in the nuclear fraction upon RNAi-mediated silencing of *FUS-DDIT3* for 72 h. GAPDH, cytoplasmic marker; Histone H3, nuclear marker. N nucleus, C cytoplasm. **E** Incubation of serum-starved MLS 1765-92 with recombinant IGF-II reverses the effects of *FUS-DDIT3* depletion on p-LATS1 (Thr1079) and p-YAP1 (Ser127; Ser397). Representative immunoblots from at least three independent experiments are shown.

To investigate if IGF-IR signaling pathway effectors and YAP1 are co-regulated in MLS development, we analyzed the expression of IGF-II, IGF-IR, and YAP1 in tumor specimens from 54 MLS patients using IHC (Fig. 1A). IGF-IR and IGF-II were detected in 50% (27/54) and 72% (39/54) of cases, respectively, and 89% (48/54) displayed nuclear YAP1 expression (Fig. 1A). Concurrent IGF-IR, IGF-II, and nuclear YAP1 immunopositivity was found in 39% (21/54) of cases (Fig. 1A). Analyzing IGF-IR-positive [+] and -negative [−] cases separately, we observed that nuclear YAP1 was co-expressed in 96.3% (26/27) of IGF-IR [+] and 81.5% (22/27) of IGF-IR [−] MLS. To evaluate if YAP1 expression and activation of the IGF-IR/PI3K/AKT pathway are jointly dependent on FUS-DDIT3, we analyzed the effects of FUS-DDIT3 overexpression in SCP-1 cells. Immunoblotting revealed that FUS-DDIT3 expression led to (i) activation of IGF-IR/PI3K/AKT signaling, as evidenced by increased IGF-IR (Tyr1135/1136) and AKT (Ser473) phosphorylation; (ii) decreased phosphorylation of the Hippo effectors LATS1 and MOB1 (Thr1079 and Thr35, respectively); and (iii) increased expression of YAP1 and its downstream targets FOXM1 and Survivin (Fig. 1B). Accordingly, RNAi-mediated silencing of *FUS-DDIT3* in MLS 1765-92 resulted in deactivation of the IGF-IR/PI3K/AKT cascade, as shown by decreased IGF-IR expression associated with a strong reduction of phosphorylated AKT (Ser473) (Fig. 1C). Consistently, *FUS-DDIT3* knockdown resulted in enhanced phosphorylation of LATS1 (Thr1079) and MOB1 (Thr35) and decreased expression of YAP1, FOXM1, and Survivin (Fig. 1C). Subcellular fractionation of MLS 1765-92 revealed that *FUS-DDIT3* depletion caused increased phosphorylation of YAP1 (Ser127), which was particularly evident in the cytoplasm and diminished nuclear YAP1 levels, corresponding to a decrease in transcriptionally active YAP1 (Fig. 1D). Notably, YAP1 inactivation upon *FUS-DDIT3* depletion could be reversed by external addition of IGF-II (Fig. 1E). Together, these results show that FUS-DDIT3 is involved in the concurrent activation of IGF-IR-dependent signals and nuclear expression of YAP1 in MLS cells.

### IGF-IR/PI3K/AKT-mediated signals promote YAP1 activity

**Fig. 2:**
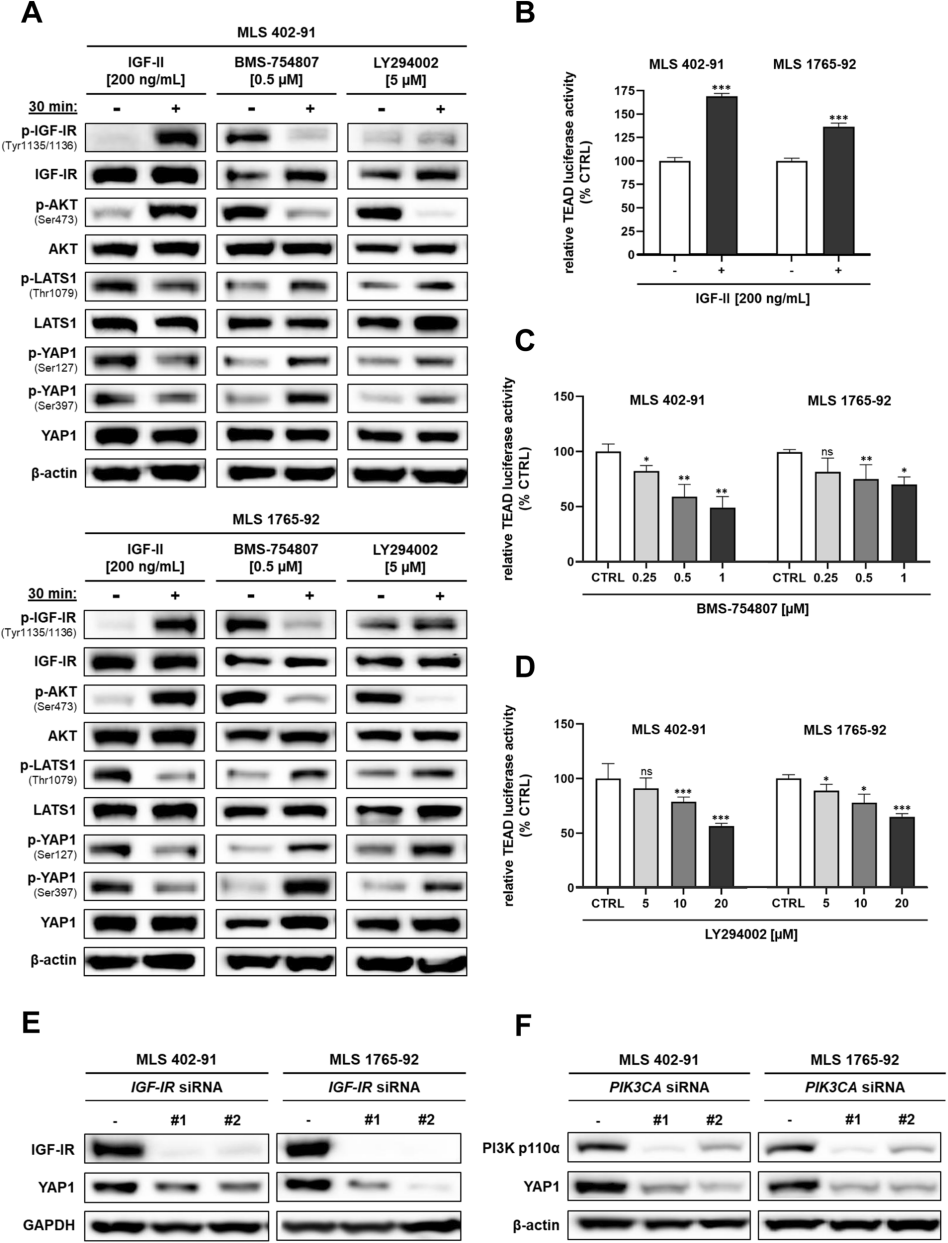
IGF-IR/PI3K/AKT-mediated regulation of YAP1 in MLS. **A** IGF-II stimulation of starved MLS 402-91 and MLS 1765-92 cells activates the IGF-IR/PI3K/AKT cascade, associated with reduced p-LATS1 (Thr1079), p-YAP1 (Ser127), and p-YAP1 (Ser397) protein levels. Treatment with BMS-754807 (IGF-IR inhibitor) or LY294002 (PI3K inhibitor) for 30 min shows inverse effects. **B** IGF-II stimulation of starved MLS 402-91 and MLS 1765-92 cells for 6 h significantly increases TEAD luciferase reporter activity (error bars represent the mean + SD of three independent experiments; unpaired *t* test, ***P < 0.001). **C**, **D** Treatment with BMS-754807 or LY294002 leads to a dose-dependent reduction of TEAD luciferase reporter activity (error bars represent the mean + SD of three independent experiments, unpaired *t* test; *P < 0.05, **P < 0.01, ***P < 0.001); ns, not significant. **E**, **F** Decreased YAP1 levels upon RNAi-mediated silencing of *IGF-IR* or *PIK3CA* for 72 h compared to non-targeting control siRNA. Representative immunoblots of at least three independent experiments with similar results are shown.

Assuming cross-connections between these different signaling pathways, we further investigated the involvement of FUS-DDIT3-driven IGF-IR/PI3K/AKT signals in Hippo/YAP1 deregulation in MLS cells. Treatment of serum-starved MLS cell lines with IGF-II resulted in enhanced phosphorylation of IGF-IR (Tyr1135/1136) and AKT (Ser473), reduced phosphorylation of LATS1 (Thr1079), and diminished phosphorylation of YAP1 (Ser127, Ser397) (Fig. 2A). To verify that the IGF-IR/PI3K/AKT axis modulates YAP1 activity, we investigated the effects of the IGF-IR antagonist BMS-754807 and the PI3K inhibitor LY294002 on the Hippo/YAP1 signaling axis. Immunoblots demonstrated that inhibition of IGF-IR or PI3K suppresses the phosphorylation of AKT (Ser473) while augmenting the phosphorylation of LATS1 (Thr1079) and YAP1 (Ser127, Ser397). To confirm the effects on YAP1 activity, TEAD luciferase reporter assays were conducted, demonstrating that the changes in YAP1 phosphorylation upon IGF-II stimulation were associated with increased transcriptional activity of YAP1 (Fig. 2B). Conversely, IGF-IR or PI3K inhibition resulted in decreased transcriptional activity (Fig. 2C, D). Consistent with the effects of pharmacologic inhibition, RNAi-mediated silencing of *IGF-IR* or *PIK3CA* led to decreased YAP1 protein levels (Fig. 2E, F). Collectively, these results indicate that the IGF-IR/PI3K/AKT signaling axis contributes to the deregulation of Hippo/YAP1 signaling in MLS.

### FUS-DDIT3 and YAP1/TEAD co-localize in the nucleus

**Fig. 3:**
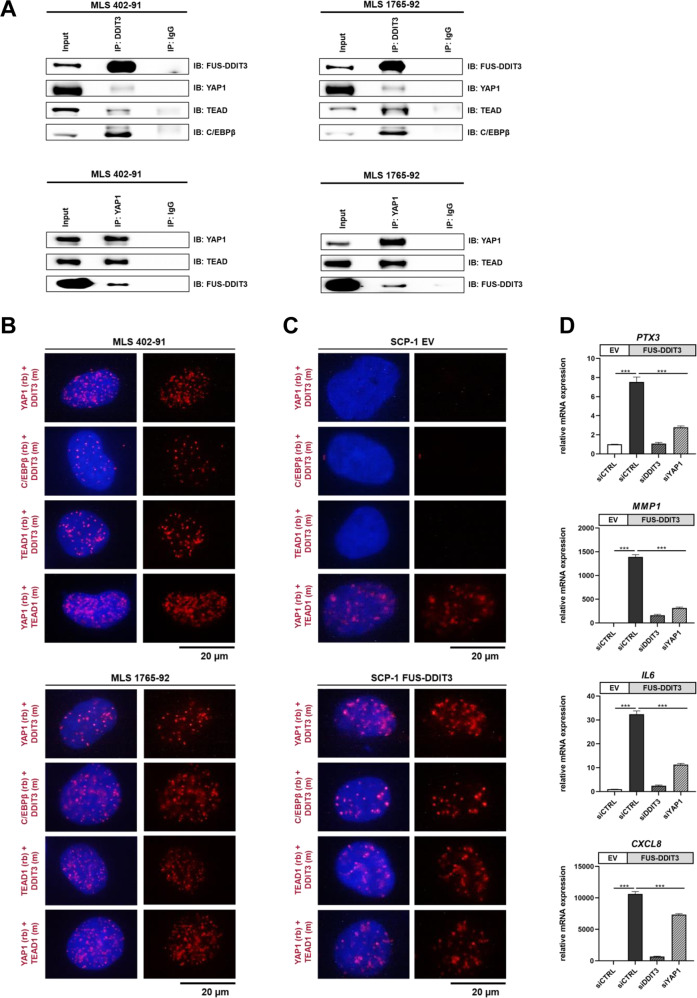
Nuclear interaction between FUS-DDIT3 and YAP1/TEAD. **A** Reciprocal co-immunoprecipitation experiments with nuclear protein extracts confirm an interaction between FUS-DDIT3 and YAP1/TEAD in MLS 402-91 and MLS 1765-92 cells. The known FUS-DDIT3 interaction partner C/EBPβ served as a positive control for FUS-DDIT3. TEAD was used as a positive control for YAP1. Representative immunoblots of three independent experiments with similar results are shown. **B** PLAs validate the association of FUS-DDIT3 with YAP1, TEAD, and C/EBPβ in MLS cell lines. Red signals indicate close proximity between proteins of interest. Nuclei were counterstained with DAPI (blue; original magnification ×63 oil). One of at least two independent experiments with similar results is shown. **C** PLAs conducted in SCP-1 cells stably expressing FUS-DDIT3 or EV. One of at least two independent experiments with similar results is shown. **D** RT-qPCR analyses showing mRNA levels of selected FUS-DDIT3 target genes in SCP-1 FUS-DDIT3 or EV cells. RNAi-mediated knockdown of both *FUS-DDIT3* and *YAP1* for 48 h led to reduced expression of *PTX3*, *MMP1*, *IL6*, and *CXCL8* in SCP-1 FUS-DDIT3 cells. All mRNA levels were normalized to *GAPDH*. Data are representative of three independent experiments and presented as the mean of triplicate values SD; ***P < 0.001.

To further explore whether nuclear YAP1 plays a role in FUS-DDIT3-mediated transcriptional dysregulation, we performed co-immunoprecipitation experiments with nuclear extracts of MLS 402-91 and MLS 1765-92, demonstrating an interaction between FUS-DDIT3, YAP1, and TEAD transcription factors (Fig. 3A). C/EBPβ, a known interaction partner of FUS-DDIT3 [5, 19], was included as positive control. PLAs confirmed the nuclear interaction of FUS-DDIT3, YAP1, C/EBPβ, and TEAD in MLS cells (Fig. 3B) as well as in FUS-DDIT3-expressing SCP-1 cells (Fig. 3C). Based on these results, we investigated whether FUS-DDIT3 and YAP1 share a common transcriptional program. RT-qPCR analysis of SCP-1 cells demonstrated FUS-DDIT3-dependent upregulation of known FUS-DDIT3 target genes [20–23], i.e. *PTX3, MMP1, IL6*, and *CXCL8*. Conversely, RNAi-mediated silencing of *FUS-DDIT3* or *YAP1* reduced mRNA levels of these genes in FUS-DDIT3-expressing SCP-1 cells (Fig. 3D, Supplementary Fig. S1B), implying a combined action of FUS-DDIT3 and YAP1 in transcriptional dysregulation. Together, these results document physical interaction of FUS-DDIT3 and YAP1/TEAD in the nucleus of MLS cells, and imply that YAP1 is involved in the co-regulation of a set of FUS-DDIT3 target genes.

### FUS-DDIT3 and YAP1 co-regulate oncogenic gene sets in MLS

**Fig. 4:**
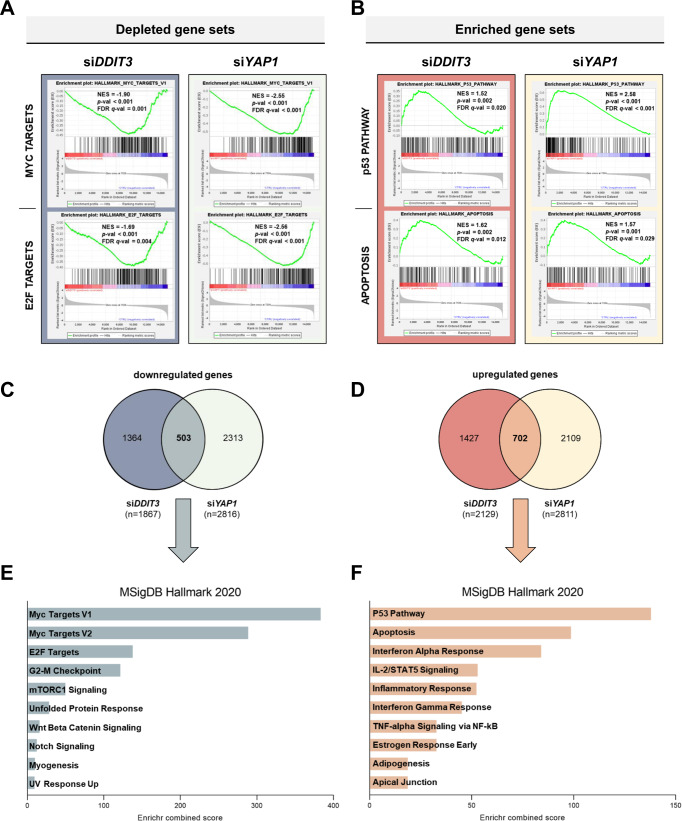
RNA-seq analysis of MLS 402-91 cells upon RNAi-mediated silencing of *FUS-DDIT3* and *YAP1*. **A** GSEA plots illustrate top depleted ‘Hallmark’ gene sets shared by the si*DDIT3* and si*YAP1* conditions, showing downregulation of MYC and E2F targets in MLS 402-91 cells. **B** Significantly enriched gene sets co-regulated by FUS-DDIT3 and YAP1 are associated with apoptosis and p53 pathways. The normalized enrichment score, nominal *P* value and false discovery rate *q*-value are shown. **C** In total, 1867 differentially expressed genes were significantly downregulated in the si*DDIT3* condition, 2816 genes were downregulated in the si*YAP1* condition, and the overlap of both conditions comprised 503 genes. **D** The set of shared upregulated genes between the si*DDIT3* (*n* = 2129) and si*YAP1* (*n* = 2811) samples comprised 702 genes. **E**, **F** Enrichr analysis of si*DDIT3*- and si*YAP1*-co-regulated genes showing the top down- and upregulated pathways derived from MSigDB Hallmark 2020.

To assess cooperative functions of FUS-DDIT3 and YAP1 reflected by co-regulated gene sets, we performed RNA-seq of MLS 402-91 cells treated with siRNAs targeting *FUS-DDIT3* or *YAP1*. Upon *FUS-DDIT3* suppression (si*DDIT3*), 3996 genes showed significantly different expression compared to the control siRNA (si*CTRL*), with 1867 genes being downregulated and 2129 genes upregulated (Fig. 4C, D). RNAi-mediated depletion of *YAP1* (si*YAP1*) resulted in differential expression of a total of 5627 genes, with 2816 genes being downregulated and 2811 genes upregulated (Fig. 4C, D, Supplementary Fig. S2A). To identify specific biological processes and pathways affected by RNAi-mediated silencing of *FUS-DDIT3* or *YAP1* in MLS 402-91, we performed gene set enrichment analysis (GSEA). Compared to si*CTRL*, si*DDIT3* and *siYAP1* cells were characterized by significant underrepresentation of 4/50 and 12/50 hallmark gene sets, respectively. For both conditions, HALLMARK_MYC_TARGETS_V2, HALLMARK_MYC_TARGETS_V1, HALLMARK_E2F_TARGETS, and HALLMARK_G2M_CHECKPOINT were the most significantly downregulated gene sets (Fig. 4A, Supplementary Table S1), highlighting the integral role of FUS-DDIT3 and YAP1 in cell proliferation and cell cycle progression. In contrast, 24/50 and 15/50 hallmark gene sets were significantly overrepresented in si*DDIT3* and si*YAP1* cells, respectively. Selected gene sets enriched in both conditions included HALLMARK_P53_PATHWAY, HALLMARK_APOPTOSIS, and HALLMARK_INFLAMMATORY_RESPONSE (Fig. 4B, Supplementary Table S1), pointing to a role of FUS-DDIT3 and YAP1 in promoting cell survival and regulation of the inflammatory response. These findings were in line with the results of fast preranked GSEA (Supplementary Fig. S2B). Assessment of the overlap of differentially expressed genes between the si*DDIT3* and si*YAP1* conditions revealed that 503 genes were jointly downregulated and 702 genes were jointly upregulated (Fig. 4C, D). To functionally analyze the overlap of deregulated genes, we employed the Enrichr tool, assigning these genes to the Molecular Signature Data Base (MSigDB) gene set library [24–26]. According to Fig. 4E, downregulated genes upon *FUS-DDIT3* and *YAP1* knockdown were enriched in MYC and E2F targets and G2M checkpoint genes, whereas upregulated gene sets were associated with the p53 pathway and apoptosis (Fig. 4F). These findings were validated by RT-qPCR analysis of selected candidate genes (Supplementary Fig. S3A). Consideration of the biological functions affected by si*YAP1*/si*DDIT3* revealed shared upregulation of genes associated with adipogenesis, which were again validated by RT-qPCR (Supplementary Fig. S3B), implying a potential role for YAP1 in FUS-DDIT3-mediated inhibition of terminal adipocytic differentiation [7]. Together, these results indicate that FUS-DDIT3 and YAP1 deregulate a shared set of genes involved in various oncogenic processes as well as inhibition of adipocyte maturation.

### FUS-DDIT3 and YAP1 cooperate to disrupt terminal adipogenic differentiation

**Fig. 5:**
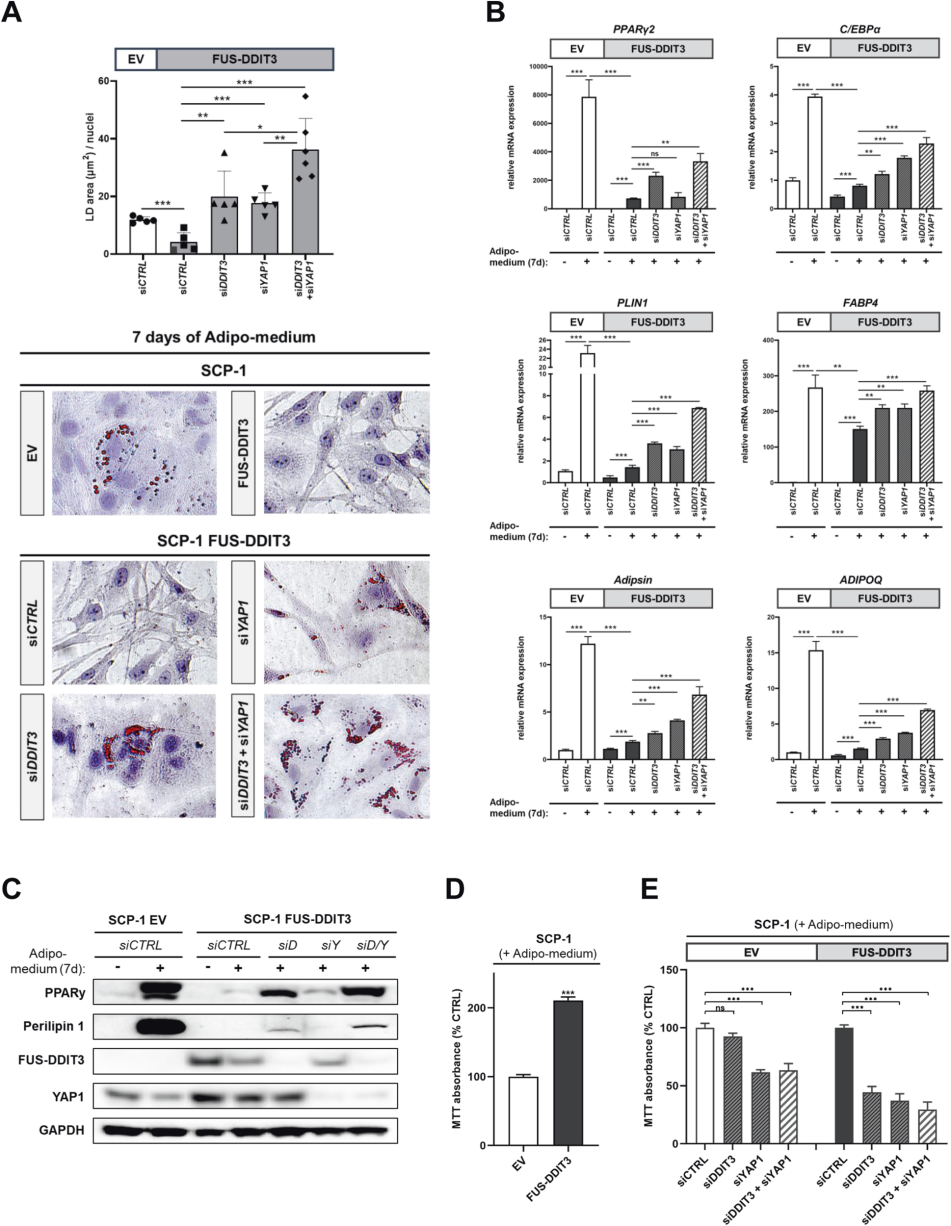
RNAi-mediated depletion of *FUS-DDIT3* and *YAP1* reverses the adipogenic differentiation arrest of FUS-DDIT3-expressing mesenchymal stem cells. SCP-1 cells stably expressing FUS-DDIT3 or EV were cultured with the indicated siRNAs with or without adipogenic differentiation medium for 7 days. **A** Lipid droplet (LD) area was quantified and divided by the number of nuclei from at least five images (×40). Data are presented as the mean + SD (*P < 0.05, **P < 0.01, ***P < 0.001). Representative images of Oil Red O staining assessing the formation of lipid droplets. Hematoxylin was used for counterstaining. One of at least three independent experiments with similar results is shown. **B** RT-qPCR results of the late stage adipogenic transcription factors *PPARγ2* and *C/EBPα*, as well as the adipogenic markers *FABP4*, *PLIN1*, *Adipsin*, and *ADIPOQ*. All mRNA levels were normalized to *GAPDH*. Data are representative of at least three independent experiments and presented as the mean of triplicate values + SD (*P < 0.05, **P < 0.01, ***P < 0.001; ns, not significant). **C** Immunoblot analysis showing the induction of PPARγ and Perilipin 1 after 7 days of incubation with indicated siRNAs with or without adipogenic differentiation medium. si*D*, *DDIT3* siRNA; si*Y*, *YAP1* siRNA. A representative immunoblot of at least three independent experiments with similar results is shown. **D** MTT proliferation assay of SCP-1 EV and FUS-DDIT3 cells exposed to adipogenic differentiation medium for 7 days (*n* = 3, mean + SD; ***P < 0.001). **E** MTT proliferation assay of SCP-1 EV and FUS-DDIT3 cells incubated with siRNAs targeting *DDIT3*, *YAP1*, or a combination of both for 7 days under differentiating conditions (*n* = 4, mean + SD; ***P < 0.001; ns, not significant).

To further investigate the role of FUS-DDIT3 in adipocytic differentiation, we incubated SCP-1 mesenchymal stem cells expressing FUS-DDIT3 or EV in adipogenic differentiation medium for 7–10 days. SCP-1 EV cells formed Oil Red O-positive lipid droplets upon induction of adipogenic differentiation, whereas this process was impaired in FUS-DDIT3-expressing SCP-1 cells (Fig. 5A, Supplementary Fig. S4). Treatment of SCP-1 FUS-DDIT3 cells with siRNAs targeting *DDIT3* restored lipid droplet formation, confirming that FUS-DDIT3 interferes with adipogenic differentiation. We then asked whether YAP1 also affects adipogenic differentiation of SCP-1 cells. RNAi-mediated depletion of *YAP1* resulted in the accumulation of lipid droplets, and this effect was enhanced upon combined silencing of *FUS-DDIT3* and *YAP1* (Fig. 5A, Supplementary Fig. S4). RT-qPCR analyses validated these observations, showing that the mRNA levels of the late-stage adipogenic master regulators *PPARγ2* and C/*EBPα*, as well as the adipogenic markers *PLIN1*, *FABP4*, *Adipsin*, and *ADIPOQ* were significantly induced in SCP-1 EV cells exposed to adipogenic medium. In contrast, the expression of these genes was attenuated in the presence of FUS-DDIT3 (Fig. 5B). This effect was partly reverted upon RNAi-mediated depletion of *FUS-DDIT3* or *YAP1* with combined silencing of *FUS-DDIT3* and *YAP1* yielding higher mRNA levels than *YAP1/FUS-DDIT3* single knockdown. Notably, silencing of *FUS-DDIT3* led to increased transcription of *PPARγ2*, whereas silencing of *YAP1* did not affect *PPARγ2* expression but was associated with elevated mRNA levels of C/*EBPα*. Accordingly, immunoblot analysis revealed that expression of PPARγ and Perilipin 1 (encoded by *PLIN1*) were markedly increased in SCP-1 EV cells upon incubation with adipogenic medium for 7 days, while this induction was significantly impaired in SCP-1 FUS-DDIT3 cells (Fig. 5C). However, transfecting SCP-1 FUS-DDIT3 cells with *DDIT3* siRNA before adipogenic induction stimulated the expression of PPARγ and Perilipin 1. Incubating cells with *YAP1* siRNA resulted in a slight increase of PPARy protein levels compared to the control, while Perilipin 1 levels were unaffected. In agreement with the RT-qPCR results, the combined knockdown of *FUS-DDIT3* and *YAP1* resulted in enhanced expression of PPARγ and Perilipin 1 at the protein level compared to single knockdown.

As cell proliferation and differentiation often demonstrate an inverse relationship, we analyzed the effect of adipogenic stimulation on proliferation. MTT assays revealed a two-fold higher proliferation rate of SCP-1 FUS-DDIT3 cells than SCP-1 EV cells cultured in adipogenic differentiation medium for 7 days (Fig. 5D). Accordingly, treating SCP-1 FUS-DDIT3 cells with *DDIT3* or *YAP1* siRNA significantly decreased cell proliferation by more than 50%, and combined depletion of *FUS-DDIT3* and *YAP1* led to an even stronger reduction of cell proliferation (Fig. 5E). In SCP-1 EV cells, *YAP1* depletion had a less pronounced anti-proliferative effect, pointing to a particular dependence of FUS-DDIT3-expressing cells on YAP1. Collectively, these data indicate that both FUS-DDIT3 and YAP1 inhibit adipogenic differentiation in FUS-DDIT3-driven cells. The shown impact of FUS-DDIT3 and YAP1 on the expression of the adipocyte master transcription factors *PPARγ2* and *C/EBPα* and the adipogenic markers supports the concept of a joint function of the fusion protein and YAP1 in MLS tumorigenesis.

## Discussion

The FUS-DDIT3 oncoprotein plays an essential role in MLS tumorigenesis [7–9], but its mode of action remains incompletely understood. Previous work identified the transcriptional co-regulator YAP1, whose activity is restricted by the Hippo signaling pathway, as a crucial driver of MLS [15]. Various mechanisms of YAP1 activation have been described in mesenchymal tumors, including (i) (epi-)genetic deregulation of Hippo pathway tumor suppressors [27]; (ii) Hippo pathway inhibition, as reported in alveolar rhabdomyosarcoma in which the PAX3-FOXO1 fusion-mediated upregulation of RASSF4 inhibits MST1 [28]; and (iii) involvement of *YAP1* in chromosomal translocations in epithelioid hemangioendothelioma (*YAP1-TFE3*) and sclerosing epithelioid fibrosarcoma (*YAP1-KMT2A*) [29, 30]. In MLS, however, recurrent genetic alterations affecting Hippo pathway components have not been identified, pointing to a mechanism of nuclear YAP1 stabilization and activation that likely depends on FUS-DDIT3 and may involve oncogenic signaling cascades known to be active in MLS, such as the IGF-II/IGF-IR/PI3K/AKT axis [12–14].

In this study, we aimed to decipher whether IGF-IR/PI3K/AKT signaling contributes to aberrant YAP1 activation and to unravel potential cooperative roles of FUS-DDIT3 and YAP1 in MLS tumorigenesis, given previous hints at their nuclear co-localization [15]. Our IHC analysis of human MLS specimens provided first evidence of a potential link between the IGF-IR and YAP1, as nuclear YAP1 was detected in almost all IGF-IR [+] cases. Functionally, we demonstrated that modulation of the IGF-IR/PI3K/AKT pathway profoundly affects Hippo/YAP1 in MLS cells via IGF-IR/PI3K-transmitted inhibition of LATS1 activity. These findings are in agreement with our previous study in synovial sarcoma, another fusion-driven soft tissue sarcoma, in which we demonstrated that SS18-SSX-mediated IGF-IR signaling acts as a critical upstream modulator of YAP1/TAZ via deregulation of the Hippo upstream effectors LATS1 and MOB1 [31]. Few reports of a regulatory IGF-IR/YAP1 axis have been published so far [32–34]: Zhou and colleagues reported an analogous mechanistic link in diffuse large B-cell lymphoma, demonstrating that IGF-IR inhibition decreased YAP1 expression and restrained the activation of YAP1 downstream targets [34]. Further evidence for the positive regulation of YAP1 by PI3K signaling was reported in mammary tumorigenesis and hepatocellular carcinoma [35–37]. Consistent with these findings, our data demonstrate that PI3K acts as an upstream regulator of YAP1 in MLS. Although IGF-IR profoundly impacts YAP1 expression, PI3K acts as a central signaling hub that integrates multiple upstream receptor tyrosine kinase signals active in MLS [38], explaining YAP1 nuclear localization in IGF-IR [-] MLS specimens. Our results provide the first mechanistic concept of aberrant YAP1 activation in MLS and may be of therapeutic relevance, particularly in advanced-stage tumors, which are associated with a high prevalence of IGF-IR expression and activating mutations in the PI3K/AKT axis [13].

Based on our previous observation that FUS-DDIT3 expressing cells require YAP1 to survive and proliferate [15], we wondered if both oncoproteins might cooperate in driving MLS tumorigenesis. Co-immunoprecipitation experiments and PLAs in MLS in vitro systems demonstrated a nuclear interaction between FUS-DDIT3, YAP1, and TEAD transcription factors. Although YAP1 associates with different transcription factors in various contexts, TEAD transcription factors are considered the predominant mediators of YAP1 signals [16]. We thus wondered if FUS-DDIT3 mainly plays a pioneer role in the activation of YAP1/TEAD signals in MLS cells, or if YAP1 might be required for the transcriptional dysregulation mediated by FUS-DDIT3. Although there is limited data on direct FUS-DDIT3 target genes, previous studies reported that FUS-DDIT3 binds to the promoter of *PTX3* and is involved in the deregulation of NF-κB- and C/EBP-controlled genes, including *IL6*, *CXCL8*, and *MMP1* [21–23]. Our RT-qPCR data suggest that YAP1 is indeed required for the expression of genes upregulated by FUS-DDIT3, such as *PTX3*, *IL6, CXCL8*, and *MMP1*, implying a cooperative interaction of YAP1 and FUS-DDIT3 in transcriptional regulation in MLS. This prompted us to explore whether FUS-DDIT3 and YAP1 act together in establishing a coordinated gene expression program in MLS. RNA-seq of MLS cells unveiled that FUS-DDIT3 and YAP1 co-regulate oncogenic gene sets implicated in the upregulation of MYC and E2F targets and G2-M cell cycle progression while inhibiting the expression of genes associated with apoptosis, p53 pathway and interferon alpha response. Consistently, an approximate comparison of transcriptional signatures regulated by IGF-IR overexpression in human epithelial cells revealed a downregulation of gene sets involved in p53 pathway, apoptosis, and interferon alpha response (Supplementary Fig. S5), constituting an overlap with the FUS-DDIT3/YAP1-dependent signature we identified in MLS [39]. In addition, in our RNA-seq analysis genes involved in adipogenesis were enriched in the shared gene set of *FUS-DDIT3-* and *YAP1*-depleted MLS cells, which caught our attention as one of the key properties of FUS-DDIT3 is to block adipogenic differentiation [7, 8].

The differentiation of mesenchymal progenitor cells into adipocytes is a tightly regulated process. It involves a cascade of transcription factors, including C/EBPβ and C/EBPδ, that are active during the first stages of adipocytic differentiation to subsequently induce the expression of the master regulators C/EBPα and PPARγ, which play a crucial role in terminal adipogenic differentiation [40], reflected by the induction of mature adipocyte markers such as *ADIPOQ*, *adipsin*, and *FABP4* [41]. Functional studies in FUS-DDIT3 transgenic mice, FUS-DDIT3 murine embryonic fibroblasts, and human liposarcoma cell lines previously demonstrated that FUS-DDIT3 leads to transcriptional downregulation of *C/EBPα* and *PPARγ2* by interfering with C/EBPβ activity, thereby inducing an adipogenic differentiation block [42]. Our RNA-seq and RT-qPCR analyses pointed to a function of both FUS-DDIT3 and YAP1 in suppressing adipogenic genes. We, therefore, investigated the putative role of YAP1 as a negative regulator of adipogenesis in MLS using adipogenic differentiation assays in the SCP-1 mesenchymal stem cell system. In line with previously published studies employing MLS in vitro systems, FUS-DDIT3-expressing SCP-1 cells were impaired to differentiate upon adipogenic induction [7, 21, 43]. Interestingly, the ability of SCP-1 FUS-DDIT3 cells to differentiate was restored by knockdown of *YAP1*, an effect that could be enhanced by concomitant depletion of *FUS-DDIT3*. Consistent with the concept that terminal cell differentiation usually coincides with a proliferation arrest, adipogenic differentiation upon *FUS-DDIT3* and *YAP1* knockdown was accompanied by reduced cell proliferation. Our findings are in line with recent work in preadipocytes, showing that RNAi-mediated silencing or pharmacologic inhibition of YAP1 promoted lipid accumulation and suppressed proliferation, whereas overexpression of *YAP1* inhibited preadipocyte differentiation [44, 45]. In contrast to Deng and colleagues, who showed that YAP1 regulates *PPARγ* expression in ovine preadipocytes [45], our study in FUS-DDIT3-expressing mesenchymal stem cells suggests that the expression of *PPARγ* is predominantly controlled by FUS-DDIT3, whereas the expression of *C/EBPα* appears to be modulated by YAP1. A non-canonical role of YAP1 in adipo-osteogenic differentiation was recently demonstrated in a mouse model and human mesenchymal stem cells, showing that YAP1 is implicated in osteogenic differentiation while inhibiting adipogenesis [46, 47]. Taken together, we provide evidence that YAP1, in concert with FUS-DDIT3, blocks adipogenic differentiation and accelerates proliferation of mesenchymal stem cells.

**Fig. 6:**
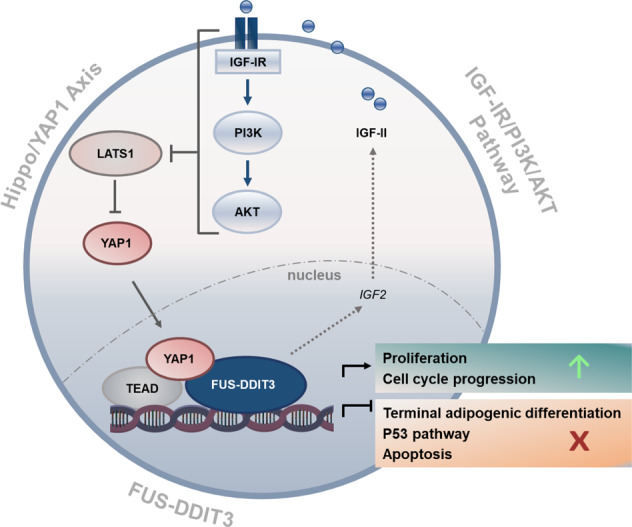
Oncogenic circuit involving FUS-DDIT3, the IGF-IR/PI3K/AKT pathway, and the Hippo/YAP1 axis in MLS. FUS-DDIT3-dependent induction of *IGF2* establishes an autocrine IGF-II/IGF-IR signaling loop, contributing to IGF-IR/PI3K/AKT pathway activation in MLS. IGF-IR and PI3K-transmitted signals interfere with the Hippo kinase LATS1, which is a direct negative regulator of YAP1, thereby promoting nuclear accumulation of transcriptionally active YAP1. In the nucleus, FUS-DDIT3 complexes with YAP1/TEAD. FUS-DDIT3 and YAP1 co-regulate oncogenic gene expression programs affecting proliferation, cell cycle progression, apoptosis, and adipogenesis.

In summary, we describe a complex oncogenic network in which FUS-DDIT3-mediated IGF-IR/PI3K/AKT signaling causes constitutive activation of YAP1, which in turn plays a fundamental role in cell proliferation and survival, co-regulates FUS-DDIT3 targets, and contributes to FUS-DDIT3-mediated blockade of adipogenic differentiation (Fig. 6). From a therapeutic point of view, IGF-IR-directed monotherapies have, thus far, shown limited effects in clinical studies involving patients with sarcoma and other types of solid tumors [48]. Next to the well-known challenges, including resistance mechanisms and the lack of solid biomarkers, the high failure rate of IGF-IR therapies is, to a large extent, due to an underestimation of the molecular diversity of IGF-IR-dependent signals. The finding that YAP1 is critically regulated through IGF-IR-mediated Hippo signals constitutes a relevant insight into this biological complexity. Refined, molecularly-based strategies integrating IGF-IR and essential downstream effectors may thus be crucial to improve the effectiveness of IGF-IR-directed therapies.

## Materials and methods

### Cell culture and cell lines

The MLS cell lines MLS 402-91 (*FUS-DDIT3* exon 7–2; type 1) and MLS 1765-92 (*FUS-DDIT3* exon 13–2; type 8) were cultured in RPMI 1640 supplemented with 10% fetal bovine serum (FBS; Gibco). The human mesenchymal SCP-1 stem cell system was previously described [15]. SCP-1 cells were cultured in MEM medium (10% FBS). All cells were grown under standard conditions (37 °C, humidified atmosphere, 5% CO_2_) and routinely tested for mycoplasma contamination by standardized PCR. Cell line identity was verified utilizing the cell authentication SNP profiling service (Multiplexion) and/or *FUS-DDIT3* gene fusion specific RT-PCR and Sanger sequencing. To study the effects of the small-molecule compounds, MLS cells were grown in medium supplemented with 2% FBS and exposed to the inhibitor for 30 min to 16 h. Before stimulation with recombinant human IGF-II (Biomol, #50342, US Biological # I7661-14), cells were starved in serum-free medium overnight.

### Tissue microarray (TMA) and immunohistochemistry (IHC)

TMAs were prepared from formalin-fixed and paraffin-embedded MLS specimens from 54 patients selected from the archive of the Gerhard-Domagk-Institute of Pathology (Münster University Hospital, Münster, Germany) to analyze the expression of IGF-IR, IGF-II, and nuclear YAP1, partly based on published data [11, 15]. Detailed information of IHC staining are described in the Supplementary Materials and methods.

### RNA interference (RNAi)

For transient RNAi-mediated silencing of *IGF-IR*, *PIK3CA*, *FUS-DDIT3*, and *YAP1*, cells were transfected with a set of prevalidated small interfering RNAs (siRNAs) using Lipofectamine RNAiMAX (Invitrogen, #13778500). Detailed procedures and siRNA sequences are provided in the Supplementary Materials and methods. The knockdown efficiency of siRNAs targeting *FUS-DDIT3* and *YAP1* are depicted in Supplementary Fig. S6.

### Compounds

The IGF-IR ATP antagonist BMS-754807 (C_23_H_24_FN_9_O; CAS#: 1001350-96-4; Cat# BM0003) was purchased from Sigma Aldrich and dissolved in DMSO (AppliChem, A3672). The pan-PI3K inhibitor LY294002 (C_19_H_17_NO_3_; CAS#: 154447-36-6; Cat# S1105) was purchased from Selleckchem and dissolved in DMSO. Final DMSO concentration did not exceed 0.2% (v/v) for all experiments.

### Cell lysate preparation and immunoblotting

Subcellular fractionation was performed using the NE-PER Nuclear and Cytoplasmic Extraction Reagents Kit (Thermo Scientific, #78835) according to the manufacturer’s instructions. Detailed information of total protein extraction, immunoblotting, and densitometric quantification (Supplementary Figs. S7, S8, and Table S2) are described in the Supplementary Materials and methods.

### Luciferase reporter assay

MLS cells were transfected with a TEAD luciferase reporter plasmid (8xGTIIC; Addgene #34615, [49]) as previously described [15]. The medium was replaced 3 h after transfection and cells were incubated in medium containing 2% FBS and the respective inhibitor. After incubation for 16 h, cells were lysed, and luciferase activity was measured in quintuplicates using the Dual-Luciferase Reporter Assay system (Promega, E1960) according to the manufacturer’s instructions. Luciferase activity was normalized to Renilla luciferase activity (co-transfected pRL-TK Renilla control plasmid, Promega).

### Nuclear co-immunoprecipitation

The Universal Magnetic Co-IP Kit (Active Motif, #54002) was used to prepare nuclear extracts from MLS cell lines according to the manufacturer’s instructions. Detailed information is provided in the Supplementary Materials and methods.

### Proximity ligation assay (PLA)

Cells were seeded in 8-well chamber slides overnight, fixed in warm 4% paraformaldehyde for 15 min at room temperature (RT), and permeabilized with 0.3% Triton X-100 for 1 h at RT. Cells were blocked with Duolink blocking solution and incubated with primary antibodies at the indicated concentrations overnight (Supplementary Table S3). Proximity ligation was performed using the Duolink PLA Red Mouse/Rabbit Kit (Sigma-Aldrich, DUO92101) according to the manufacturer’s instructions. Slides were imaged using a Leica DM5500 B microscope with a ×63 oil objective. Representative images of PLA-negative controls in MLS 402-91 are depicted in Supplementary Fig. S1A.

### RNA isolation, cDNA synthesis, and quantitative RT-PCR

Total RNA was isolated using the RNeasy Plus Mini Kit (QIAGEN, #74134), followed by cDNA synthesis with the ProtoScript II First Strand cDNA Synthesis Kit (NEB, E6560) according to the manufacturer’s instructions. Quantitative reverse transcription PCR (RT-qPCR) was performed on a StepOnePlus Real-Time PCR System (Applied Biosystems) using Power SYBR Green PCR Master Mix (Applied Biosystems, #4368708) according to the manufacturer’s instructions. Target gene expression was calculated using the ΔΔCt method and normalized to *GAPDH* and *ACTB* as reference genes. RT-qPCR primer sequences are listed in Supplementary Table S4.

### RNA sequencing (RNA-seq) and data analysis

Total RNA was purified with an RNeasy Plus Mini Kit (QIAGEN, #74134) and DNase treated. RNA concentration was determined via Qubit measurement. Samples (30 µL; concentration, 50 ng/µL) were processed by the DKFZ Genomics and Proteomics Core Facility. RNA quality was determined using an Agilent TapeStation. RNA integrity number equivalents (RINe) for all samples were measured as 10.0. Library preparation was performed using an Illumina TruSeq Stranded kit (#20020594). Each sample was individually barcoded with a pair of IDT for Illumina TruSeq RNA UD indexes. The pooled sample was distributed into two lanes of an Illumina HiSeq 4000 System, and 100 cycles of paired-end sequencing were performed. Detailed procedures for RNA-seq data processing and differential expression analysis are described in the Supplementary Materials and methods.

### Differentiation assay and Oil Red O staining

To investigate the role of FUS-DDIT3 and YAP1 in adipogenic differentiation, SCP-1 cells expressing FUS-DDIT3 or an empty control vector (EV) were incubated with complete StemPro Adipogenesis Differentiation Medium (Gibco, A1007001). Detailed procedures of the differentiation assay and Oil Red O staining are described in the Supplementary Materials and methods.

### Cell proliferation assay

The proliferation of SCP-1 FUS-DDIT3 and EV cells incubated with siRNAs and adipogenic differentiation medium for 7 days was measured using the Cell Proliferation Kit I (Roche, #11465007001) according to the manufacturer’s instructions. At least three independent experiments were performed in quintuplicates.

### Statistical analysis

Statistical analysis was conducted using Prism Software (GraphPad Prism version 9). To analyze the statistical difference between two groups, unpaired, two-tailed Student’s *t* tests were performed. Statistical differences were considered significant at *P* < 0.05 (*), *P* < 0.01 (**), and *P* < 0.001 (***). *n* indicates the numbers of independently performed experiments.

## Supplementary information

Supplementary Information

Supplementary Table S2

Supplementary Table S6

## Data Availability

RNA-seq data generated in this study has been deposited at Gene Expression Omnibus (GEO) with accession number GSE184436.
