# Supplementary material for: Fusion protein-driven IGF-IR/PI3K/AKT signals deregulate Hippo pathway promoting oncogenic cooperation of YAP1 and FUS-DDIT3 in myxoid liposarcoma
Source: Oncogenesis. 2022 Apr 22;11(1):20. doi: 10.1038/s41389-022-00394-7 (PMC9033823; doi:10.1038/s41389-022-00394-7)
Supplement: Supplementary file 1 — Supplementary Information [file 41389_2022_394_MOESM1_ESM.pdf]

Supplementary Information

Fusion protein-driven IGF-IR/PI3K/AKT signals deregulate Hippo pathway promoting oncogenic cooperation of YAP1 and FUS-DDIT3 in myxoid liposarcoma

Ruth Berthold, Ilka Isfort, Cihan Erkut, Lorena Heinst, Inga Grünewald, Eva Wardelmann, Thomas Kindler, Pierre Åman, Thomas G. P. Grünewald, Florencia Cidre-Aranaz, Marcel Trautmann, Stefan Fröhling, Claudia Scholl and Wolfgang Hartmann

Supplementary Materials and Methods

Tissue microarray (TMA) and immunohistochemistry (IHC)

TMAs were prepared from formalin-fixed and paraffin-embedded MLS specimens from 54 patients selected from the archive of the Gerhard-Domagk-Institute of Pathology (Münster University Hospital, Münster, Germany). Clinicopathological data were previously summarized (1) including only cases with complete immunohistochemical profiles. The pathognomonic translocation was detected in all cases using *DDIT3* break-apart FISH and/or RT-PCR analysis. Diagnoses were reviewed by two experienced pathologists based on current World Health Organization (WHO) criteria. Scientific analysis of the MLS cohort was approved by the Ethics Committee of the University of Münster (2015-548-f-S).

IHC staining was performed with a Bench-Mark ULTRA Autostainer (VENTANA/Roche) on 3 µm TMA sections. The staining procedure included heat-induced epitope retrieval pretreatment using Tris-Borate-EDTA buffer (pH 8.4; 95-100°C, 32-72 min) followed by incubation with respective primary antibodies for 16-120 min and signal detection employing the OptiView DAB IHC Detection Kit (VENTANA/Roche), as described previously (1). The following primary antibodies were used: YAP (monoclonal rabbit, D8H1X, 1:100, #14074, Cell Signaling Technology), IGF-IR (polyclonal rabbit, 1:100, #3027, Cell Signaling Technology), and IGF-II (monoclonal mouse, S1F2, 1:50, #05-166, Merck Millipore). Immunoreactivity was assessed using a semi-quantitative score (0, negative; 1, weak; 2, moderate; and 3, strong) defining the staining intensity in the positive control (invasive breast cancer, NST) as strong. Only tumor specimens with at least moderate staining (semi-quantitative score ≥ 2) were considered positive for the purpose of the study. The IHC readers were blinded to outcome data. The score cut-off point (positive = semi-quantitative score ≥ 2) was pre-specified without prior analyses of the clinical course.

RNA interference (RNAi)

For RNAi-mediated silencing of *FUS-DDIT3*, the C-terminal *DDIT3* portion of the fusion was targeted by a set of pre-validated siRNAs: *DDIT3* siRNA#1 (ID: VHS40605; Invitrogen), *DDIT3* siRNA#2 (ID: VHS40607; Invitrogen), and *DDIT3* siRNA#3 (5'-GGAAGUGUAUCUUCAUACAdTdT-3'), previously published as TLS-CHOP siRNA (2). Wild type *DDIT3* has been shown not to be expressed in MLS cells under standard cell culture conditions (3). To silence the expression of *IGF-IR*, *PIK3CA* and *YAP1* following siRNAs were purchased from Thermo Fisher Scientific: *IGF-IR* siRNA#1 (ID: s7211), *IGF-IR* siRNA#2 (ID: 74), *PIK3CA* siRNA#1 (ID: HSS108004), *PIK3CA* siRNA#2 (ID: HSS182305), *YAP1* siRNA#1

(ID: HSS115942), *YAP1* siRNA#2 (ID: HSS115944), *YAP1* siRNA#3 (ID: HSS173621), and a non-targeting negative control siRNA (BLOCK-iT Alexa Fluor Red Fluorescent Control, #14750100). Cells were transfected with indicated siRNAs using Lipofectamine RNAiMAX (Invitrogen, #13778500). After 48-72 h, siRNA-transfected cells were lysed, and knockdown efficiency was determined by qPCR and/or immunoblotting. Knockdown experiments shown in Fig. 1 demonstrate MLS cells transfected with an siRNA pool of *DDIT3* siRNA#1 and #3, keeping the concentration the same compared to single siRNA experiments. For the following analyses (Fig. 3-5) *DDIT3* siRNA#3 and *YAP1* siRNA#2 were applied, as these constructs showed the highest knockdown efficiency as depicted in Supplementary Fig. S6.

Cell lysate preparation and immunoblotting

For total protein extracts, cells were incubated with lysis buffer (150 mM NaCl, 1% NP-40, 50 mM Tris-HCl, pH 8.0) or 1x RIPA Buffer (Cell Signaling Technology, #9806) supplemented with 1x protease inhibitor cocktail (Roche) and phosphatase inhibitor cocktail (Roche) for 30 min on ice, followed by a centrifugation step for 10-30 min at 4°C. Protein concentration was quantified by Bradford assay. Protein extracts were subjected to SDS-PAGE and transferred to nitrocellulose membranes. Membranes were blocked with 5% dry milk or 5% BSA in TBST, followed by incubation with primary and HRP-linked secondary antibodies. Chemiluminescent signals were detected by the Molecular Imager ChemiDoc System (Image Lab Software; Bio-Rad Laboratories). Densitometric quantification of the immunoblots (biological replicates) was performed using Image Lab software version 6.1 (Bio-Rad Laboratories). The intensity of each band was quantified and normalized to GAPDH or β-actin; phosphorylated proteins were normalized to their total protein levels. The results are summarized in Supplementary Table S2 and Supplementary Fig. S7 and S8.

Nuclear co-immunoprecipitation

Nuclear extracts (200-300 µg) were incubated with 10 µL YAP antibody (Cell Signaling Technology, #14074) or 4 µL DDIT3 antibody (Cell Signaling Technology, #2895) in a total volume of 500 µL overnight at 4°C on a rotator. As an isotype-specific negative control, a corresponding concentration of rabbit IgG or mouse IgG2a antibody was used (Supplementary Table S3). The next day, 25 µL of protein G magnetic beads were added, and the samples were incubated for 2 h on a rotator at 4°C. Beads were washed, nuclear complexes were eluted and subjected to immunoblotting.

Differentiation assay and Oil Red O staining

Cells were seeded into 12-well or 6-well plates with MEM medium containing 10% FBS and incubated overnight. Cells were transfected with indicated siRNAs using Lipofectamine RNAiMAX as previously described. After 6-8 h of incubation, cells were washed with 1x PBS and incubated in complete StemPro Adipogenesis Differentiation Medium (Gibco, A1007001) or control medium (MEM containing 10% FBS). The medium was changed every three to four days. After seven to ten days under differentiating conditions, cells were washed in 1x PBS and RNA/proteins were isolated as described above. For Oil Red O stains, cells were washed with 1x PBS and fixed with 10% formaldehyde for 30 min at RT. After rinsing with sterile water, 60% isopropanol was added to the cells for 5 min. Subsequently, the double-filtered Oil Red O solution (0.3% in isopropanol; $C_{26}H_{24}N_4O$; CAS#1320-06-5; Sigma Aldrich) diluted 3:2 in H_2O was added to the wells and incubated for 10 min at RT protected from light. Cells were rinsed with H_2O several times and hematoxylin counterstain ($C_{16}H_{14}O_6xH_2O$; CAS#517-28-2; Merck) was added for 30 seconds. Cells were analyzed using an Olympus IX73 inverted microscope at 40x magnification. Lipid droplet area per cell was evaluated with Image J software analysing at least five images (x40).

RNA-seq data processing

Reads from Illumina sequencing were processed with the DKFZ/ODCF RNAseq workflow 1.3.0 (https://github.com/DKFZ-ODCF/RNAseqWorkflow). First, FASTQ reads for each RNA sample were aligned via 2-pass alignment using STAR 2.5.3a (4). The STAR index was generated from the 1000 Genomes Project Phase II Reference Genome (hs37d5) based on NCBI GRCh37, and GENCODE 19 gene models. Alignment call parameters are listed in Supplementary Table S5. Other parameters were as default or only pertinent for specific samples. Duplicate marking of the resultant main alignment file was done with sambamba 0.6.5 (5) using 8 threads. The chimeric file was sorted using samtools 1.6 (6) and then duplicates were marked using sambamba. BAM indexes were also generated using sambamba. Quality control analysis was performed using samtools flagstat (6) and the rnaseqc tool version 1.1.8 (7) with the 1000 Genomes Assembly and GENCODE 19 gene models. Depth of coverage analysis for rnaseqc was turned off. Gene-specific read counting was performed using featureCounts version 1.5.1 (8) over exon features based on the GENCODE 19 gene models. Both reads of a paired fragment were used for counting and the quality threshold was set to 255, which indicates that STAR found a unique alignment. Strand specific counting was also used. A custom script was used to calculate RPKM and TPM expression values. For total library abundance calculations, all genes on chromosomes X, Y,

MT as well as rRNA and tRNA genes were omitted, as they are likely to introduce library size estimation biases. All computations were performed on a high-performance compute cluster.

Differential expression analysis

Gene-specific read count data was imported to an R environment. Active genes were selected with the zFPKM method using the default zFPKM cutoff of -3 (9). Next, differential expression analysis was performed using DESeq2 package (10). Control samples were used as baseline for all comparisons. False discovery rate was controlled at 5% using independent hypothesis weighting (11). Misleading fold changes of genes with low expression or high dispersion were adjusted using the approximate posterior estimation method (12). For each comparison, genes with false discovery rate less than 5% and at least 1.15-fold increase or decrease were selected as significantly upregulated or downregulated genes, respectively. Results of differential gene expression analysis of RNA-seq data upon *FUS-DDIT3* and *YAP1* depletion are documented in Supplementary Table S6A and S6B. The same approach was applied for reanalysis of a published gene expression data set (GSE83083) in IGF-IR overexpressing human epithelial cells (13). All computations were performed on a designated workstation. Gene set enrichment analysis (GSEA) was performed to analyze the gene expression data by determining statistically significant differences in pre-defined gene sets between the si*CTRL* condition and the si*DDIT3* and si*YAP1* conditions using the GSEA software (version 4.1.0) applying default parameters (14). Results are shown in Supplementary Table S1. To identify enriched pathways, the pre-defined hallmark gene set ‘h.all.v7.4.symbols.gmt’ from the Molecular Signature Database (MSigDB) was analyzed (15). MSigDB enrichment analysis of the gene overlap of down- or upregulated genes was performed using Enrichr gene enrichment analysis tool (16, 17). Results were reported as combined score (multiplying the log of the p-value computed with the Fisher exact test by the z-score of the deviation from the expected rank).

Supplementary References

1. Trautmann M, Menzel J, Bertling C, Cyra M, Isfort I, Steinestel K et al. FUS-DDIT3 Fusion Protein-Driven IGF-IR Signaling is a Therapeutic Target in Myxoid Liposarcoma. Clin Cancer Res 2017; 23(20):6227–38.

2. Oikawa K, Tanaka M, Itoh S, Takanashi M, Ozaki T, Muragaki Y et al. A novel oncogenic pathway by TLS-CHOP involving repression of MDA-7/IL-24 expression. Br J Cancer 2012; 106(12):1976–9.

3. Yu JSE, Colborne S, Hughes CS, Morin GB, Nielsen TO. The FUS-DDIT3 Interactome in Myxoid Liposarcoma. Neoplasia 2019; 21(8):740–51.

4. Dobin A, Davis CA, Schlesinger F, Drenkow J, Zaleski C, Jha S et al. STAR: ultrafast universal RNA-seq aligner. Bioinformatics 2013; 29(1):15–21.

5. Tarasov A, Vilella AJ, Cuppen E, Nijman IJ, Prins P. Sambamba: fast processing of NGS alignment formats. Bioinformatics 2015; 31(12):2032–4.

6. Li H, Handsaker B, Wysoker A, Fennell T, Ruan J, Homer N et al. The Sequence Alignment/Map format and SAMtools. Bioinformatics 2009; 25(16):2078–9.

7. DeLuca DS, Levin JZ, Sivachenko A, Fennell T, Nazaire M-D, Williams C et al. RNA-SeQC: RNA-seq metrics for quality control and process optimization. Bioinformatics 2012; 28(11):1530–2.

8. Liao Y, Smyth GK, Shi W. featureCounts: an efficient general purpose program for assigning sequence reads to genomic features. Bioinformatics 2014; 30(7):923–30.

9. Hart T, Komori H, LaMere S, Podshivalova K, Salomon DR. Finding the active genes in deep RNA-seq gene expression studies. BMC Genomics 2013; 14(1):778.

10. Love MI, Huber W, Anders S. Moderated estimation of fold change and dispersion for RNA-seq data with DESeq2. Genome Biol 2014; 15(12).

11. Ignatiadis N, Klaus B, Zaugg JB, Huber W. Data-driven hypothesis weighting increases detection power in genome-scale multiple testing. Nat Methods 2016; 13(7):577–80.

12. Zhu A, Ibrahim JG, Love MI. Heavy-tailed prior distributions for sequence count data: removing the noise and preserving large differences. Bioinformatics 2019; 35(12):2084–92.

13. Rahman M, MacNeil SM, Jenkins DF, Shrestha G, Wyatt SR, McQuerry JA et al. Activity of distinct growth factor receptor network components in breast tumors uncovers two biologically relevant subtypes. Genome Med 2017; 9(1):40.

14. Subramanian A, Tamayo P, Mootha VK, Mukherjee S, Ebert BL, Gillette MA et al. Gene set enrichment analysis: a knowledge-based approach for interpreting genome-wide expression profiles. Proc Natl Acad Sci U S A 2005; 102(43):15545–50.

15. Liberzon A, Subramanian A, Pinchback R, Thorvaldsdóttir H, Tamayo P, Mesirov JP. Molecular signatures database (MSigDB) 3.0. Bioinformatics 2011; 27(12):1739–40.

16. Chen EY, Tan CM, Kou Y, Duan Q, Wang Z, Meirelles GV et al. Enrichr: interactive and collaborative HTML5 gene list enrichment analysis tool. BMC Bioinformatics 2013; 14:128.

17. Kuleshov MV, Jones MR, Rouillard AD, Fernandez NF, Duan Q, Wang Z et al. Enrichr: a comprehensive gene set enrichment analysis web server 2016 update. Nucleic Acids Res 2016; 44(W1):W90-7.

Supplementary Figure Legends

Supplementary Fig. S1. (A) Representative images of technical PLA negative controls acquired in MLS 402-91 with the same settings as the images presented in Fig. 3B. Left panel, separate omission of each primary antibody to detect non-specific binding of primary antibodies (single AB CTRL). Right panel, omission of all primary antibodies to detect non-specific binding of PLA probes (no AB CTRL). As biological negative control, the DDIT3 antibody was incubated with an N-cadherin antibody, which is a non-interacting protein of FUS-DDIT3. Original magnification, x63 oil. AB, antibody. **(B)** Validation of RT-qPCR results shown in Fig. 3D with two different siRNAs targeting *FUS-DDIT3* or *YAP1* in SCP-1 FUS-DDIT3- or EV-expressing cells, showing the mRNA levels of *PTX3*, *MMP1*, *IL6*, and *CXCL8* after 48 h. All mRNA levels were normalized to *GAPDH.* Data are presented as mean of duplicate values + SD (*$P < 0.05$, **$P < 0.01$).

Supplementary Fig. S2. (A) MA plots of differential expression analysis showing deregulated genes in MLS 402-91 cells following RNAi-mediated silencing of *FUS-DDIT3* or *YAP1* for 48 h in biological duplicates. Colors indicate the significance level (blue, $P < 0.05$; gray, $P > 0.05$). Numbers at the bottom indicate significantly up-/downregulated genes compared to siRNA *CTRL*. **(B)** Results of fast preranked GSEA of si*DDIT3* and si*YAP1* conditions.

Supplementary Fig. S3. (A) RT-qPCR analysis of selected targets was conducted to validate the RNA-seq analysis of MLS 402-91 cells, confirming the downregulation of MYC/E2F/G2M gene signatures (HALLMARK_MYC_TARGETS_V1, HALLMARK_E2F_TARGETS, HALLMARK_G2M_CHECKPOINT) and the upregulation of genes associated with the p53 pathway and apoptosis (HALLMARK_P53_PATHWAY, HALLMARK_APOPTOSIS). **(B)** RT-qPCR analysis further validated the upregulation of genes involved in adipogenesis (HALLMARK_ADIPOGENESIS) in the si*DDIT3* and si*YAP1* conditions of MLS 402-91 cells. All mRNA levels were normalized to *ACTB* expression. Data of three independent experiments are presented. Primer sequences are listed in Supplementary Table S4.

Supplementary Fig. S4. SCP-1 cells stably expressing FUS-DDIT3 or EV were cultured with the indicated siRNAs with or without adipogenic differentiation medium for seven and ten days. Representative images of Oil Red O staining assessing the formation of lipid droplets. Hematoxylin was used for counterstaining. Original magnification, 40x. Inset, 3.3x zoom.

Supplementary Fig. S5. Reanalysis of published gene expression data (GSE83083) in IGF-IR overexpressing human epithelial cells (13). Downregulated genes were analysed employing the Enrichr gene set enrichment tool.

Supplementary Fig. S6. (A) RT-qPCR analysis of MLS 402-91 cells incubated with different siRNAs (#1-#3) targeting *YAP1* or *DDIT3* for 48 h was performed to evaluate knockdown efficiency. All mRNA levels were normalized to *ACTB*. Data are presented as the mean of duplicate values + SD, $^{**}P < 0.01$, $^{***}P < 0.001$. **(B)** RNAi-mediated reduction of FUS-DDIT3 and YAP1 protein levels in MLS 402-91 cells. Total protein was extracted and subjected to immunoblot analysis 48 h after treatment with three siRNA duplex oligos targeting the DDIT3 portion of the chimeric FUS-DDIT3 oncoprotein and YAP1, respectively. β-actin was used as loading reference.

Supplementary Fig. S7. (A-D) Densitometric analysis of indicated immunoblots. Results are plotted as ratio of protein/ housekeeper or phosphorylated (p-) protein/ total (t-) protein of three independent experiments.

Supplementary Fig. S8. (A-C) Densitometric analysis of indicated immunoblots. Results are depicted as the ratio of phosphorylated (p-) protein/ total (t-) protein of three independent experiments.

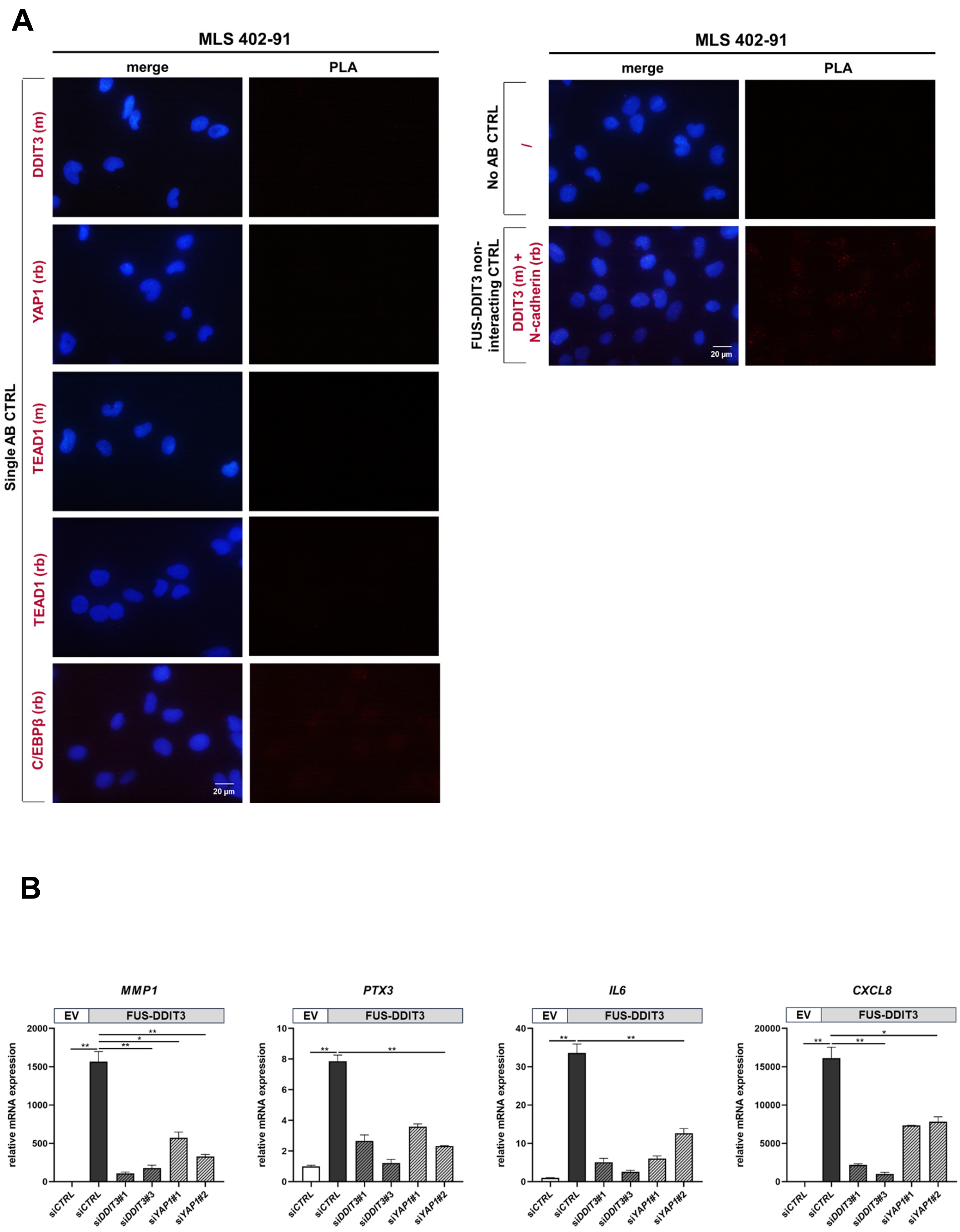

Supplementary Figure S1.

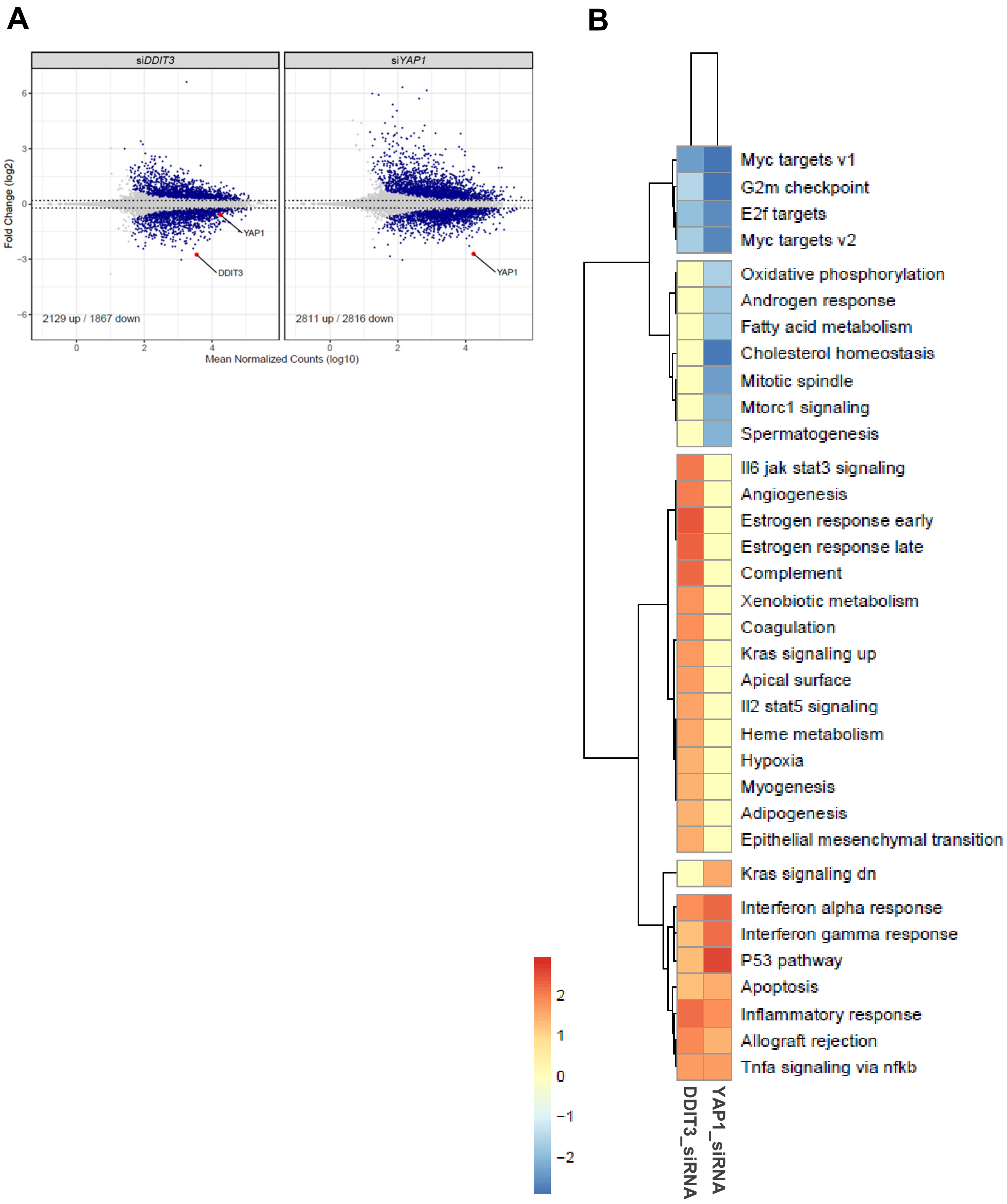

Supplementary Figure S2.

A

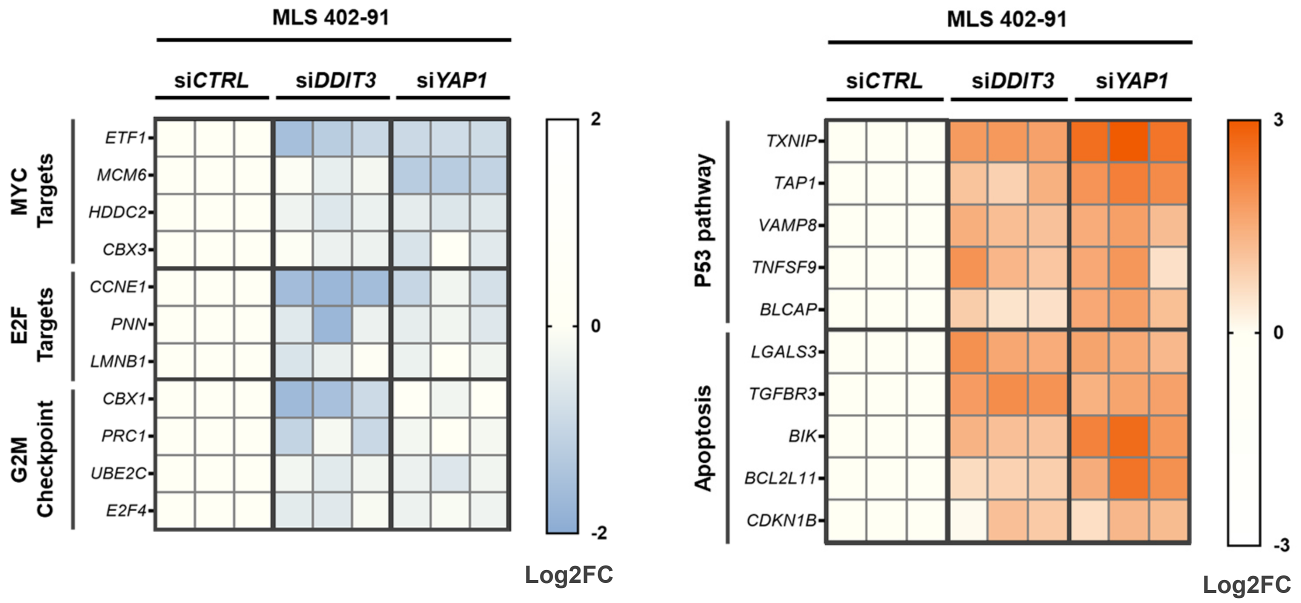

B

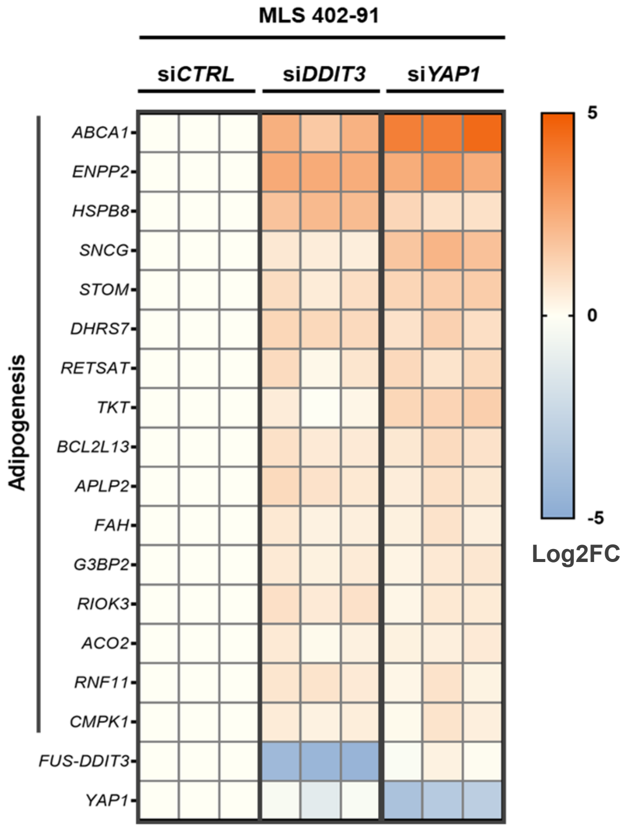

Supplementary Figure S3.

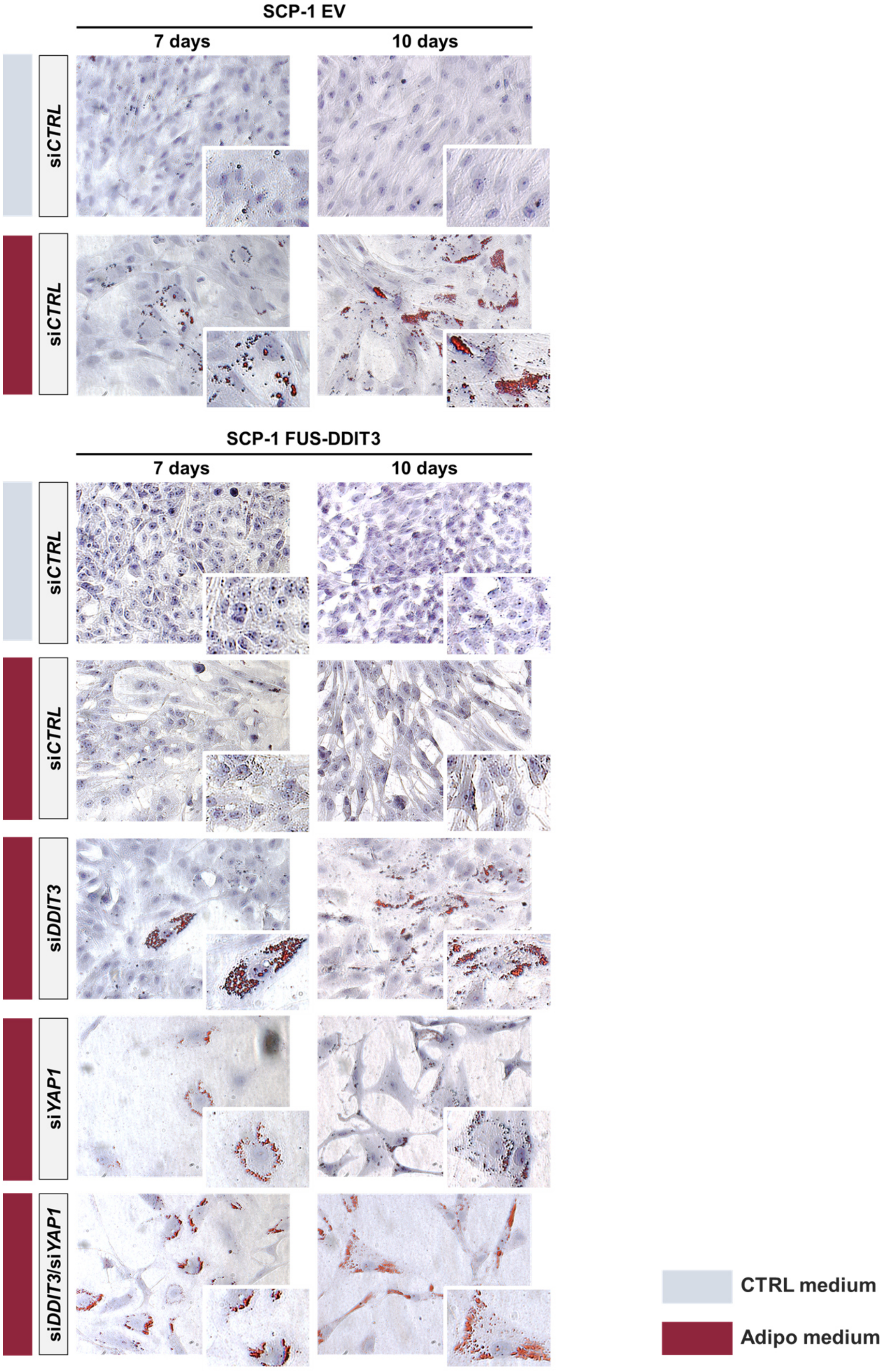

Supplementary Figure S4.

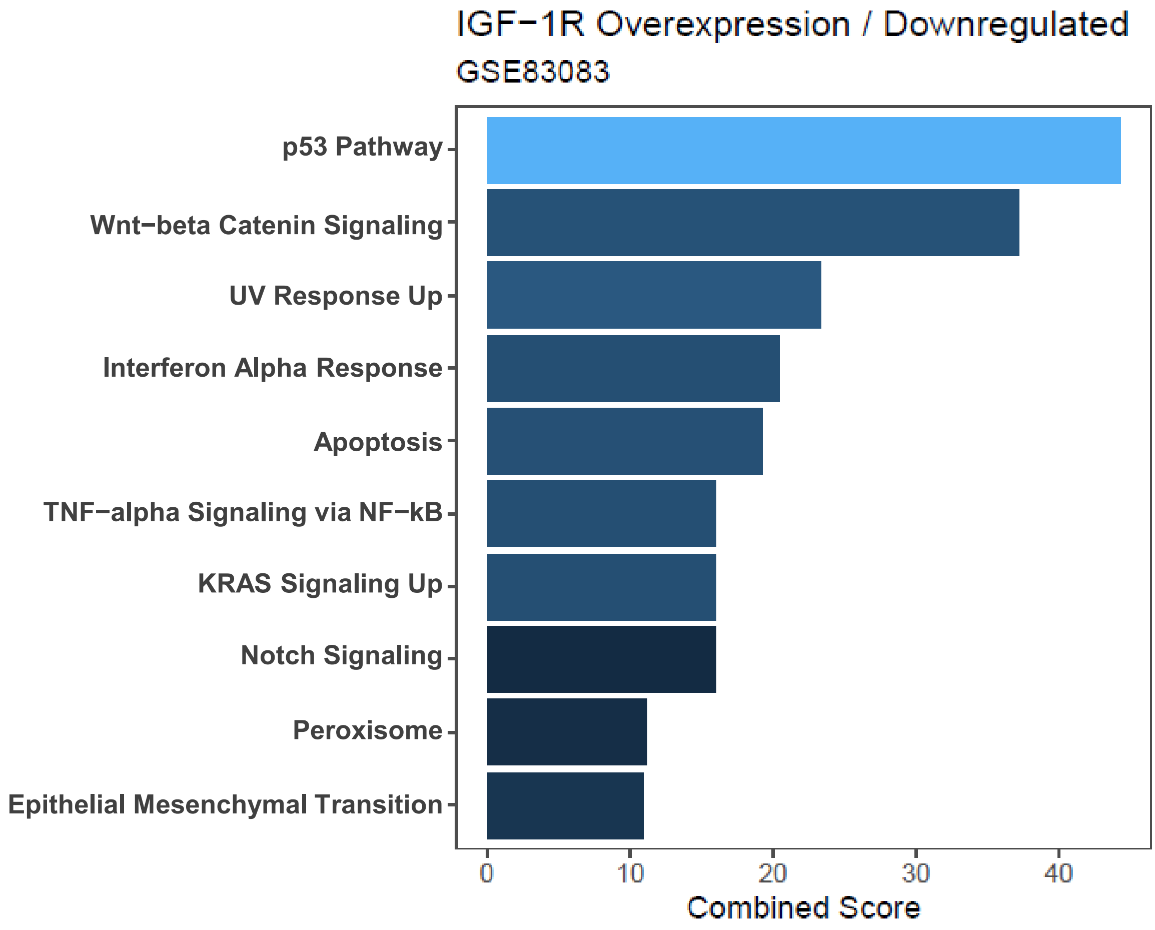

Supplementary Figure S5.

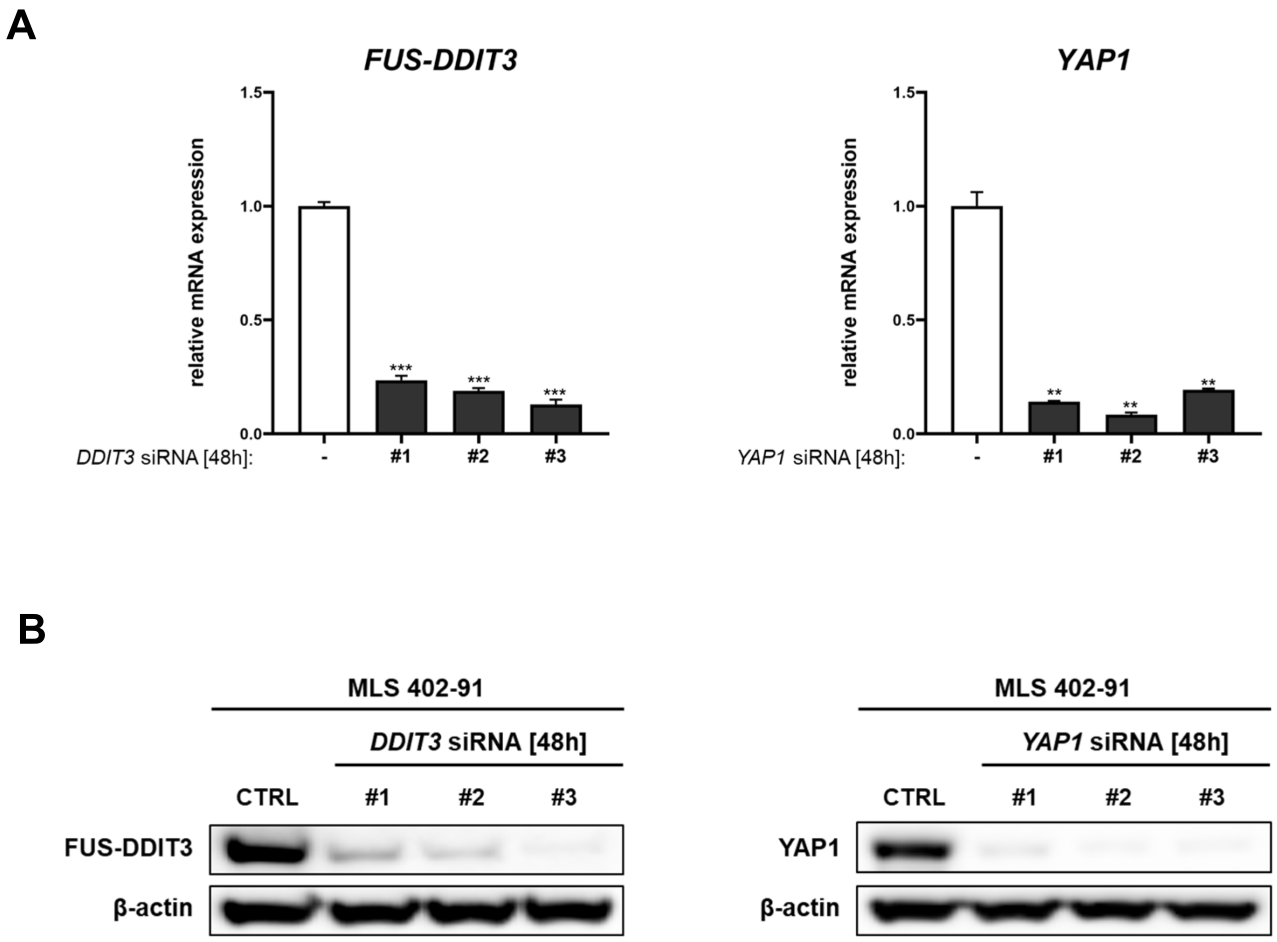

Supplementary Figure S6.

A Densitometric analysis of immunoblots shown in Fig. 1B (SCP-1 EV vs. FUS-DDIT3)

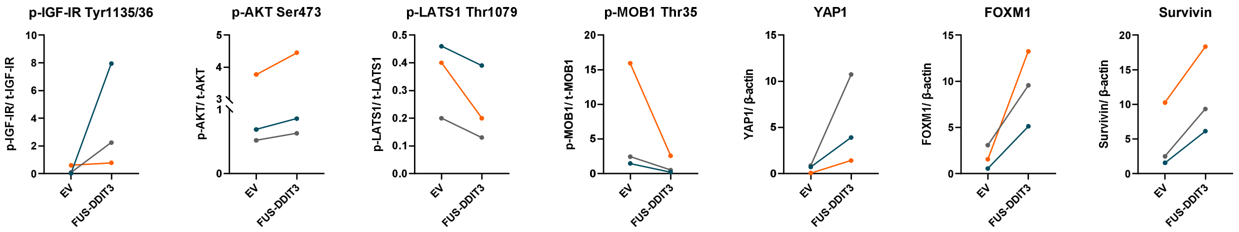

B Densitometric analysis of immunoblots shown in Fig. 1C (MLS 1765-92 -/+ si*DDIT3*; 48 h)

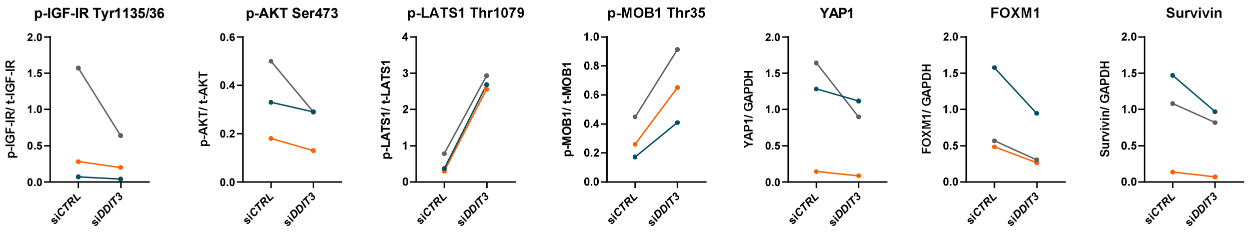

C Densitometric analysis of immunoblots shown in Fig. 1D (MLS 1765-92 -/+ si*DDIT3*; 72 h)

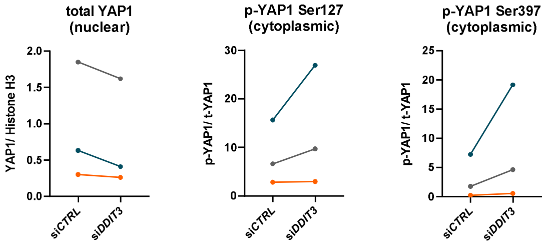

D Densitometric analysis of immunoblots shown in Fig. 1E (MLS 1765-92 -/+ si*DDIT3* [48 h]; -/+ IGF-II [0.5 h])

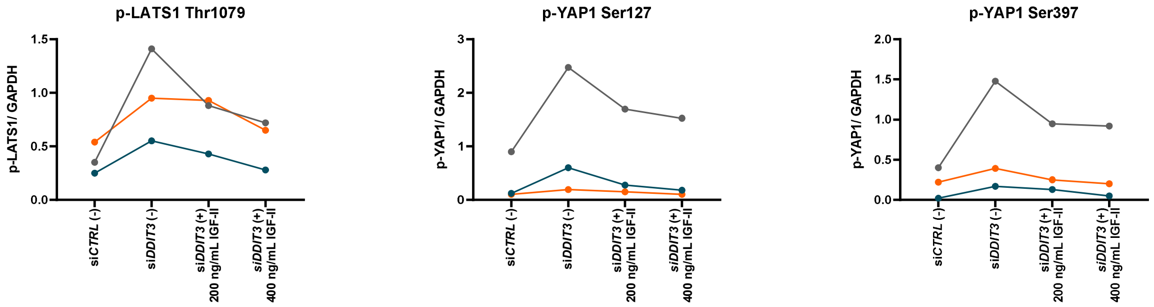

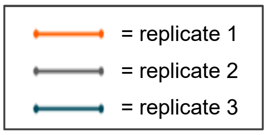

Supplementary Figure S7.

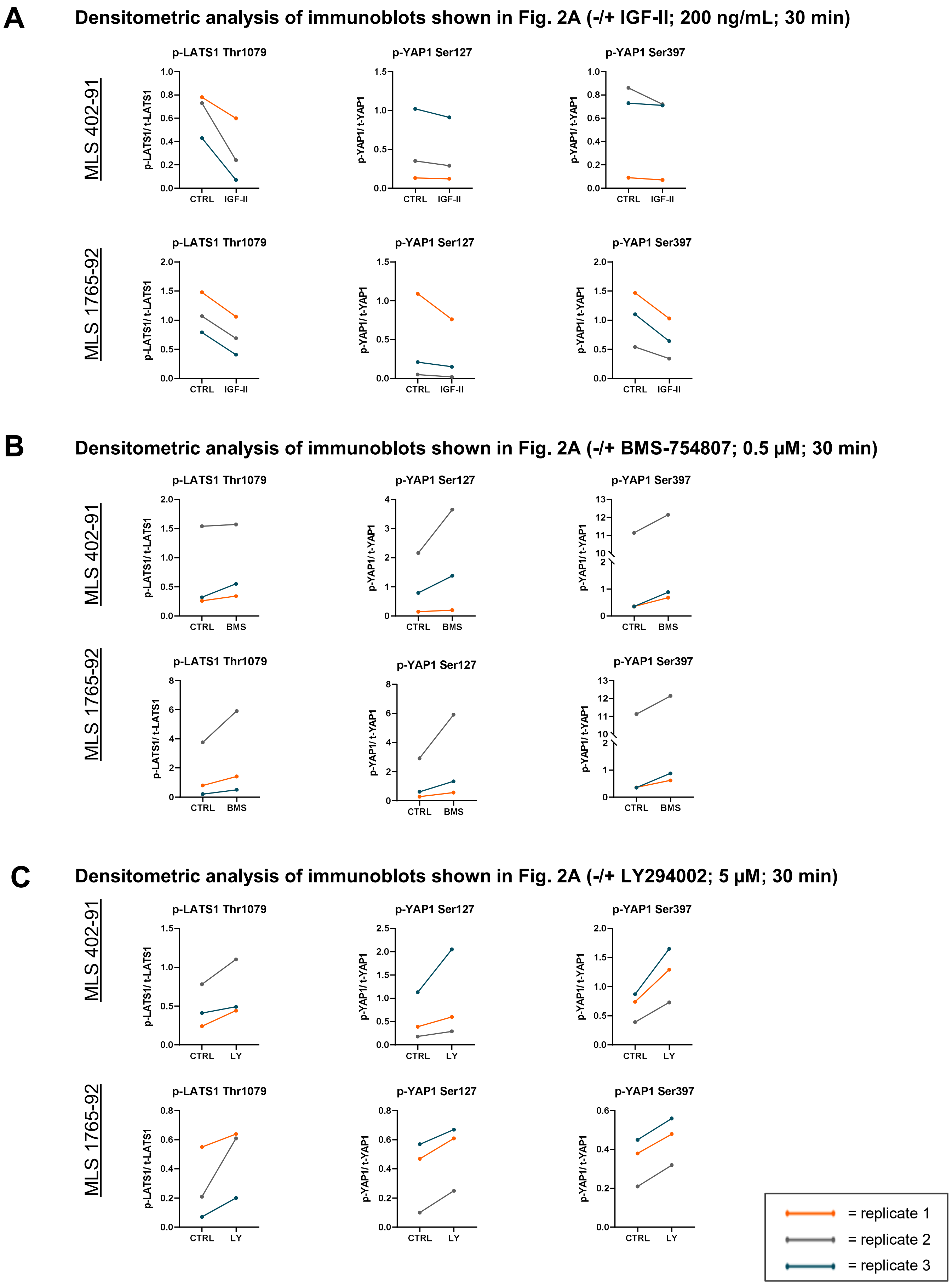

Supplementary Figure S8.

Supplementary Table S1. Gene Set Enrichment Analysis (GSEA) results. Only significant gene sets are shown (p-value < 0.05; FDR < 0.25).
ES, enrichment score; NES, normalized enrichment score; NOM p-value, nominal p-value; FDR, false discovery rate.

a. GSEA 'Hallmark gene sets' depleted in MLS 402-91 upon *FUS-DDIT3* knockdown.

	Gene set	Gene set size	ES	NES	NOM p-value	FDR q-value
1	HALLMARK_MYC_TARGETS_V2	57	-0.57	-1.99	0.000	0.000
2	HALLMARK_MYC_TARGETS_V1	199	-0.45	-1.92	0.000	0.000
3	HALLMARK_E2F_TARGETS	200	-0.39	-1.69	0.000	0.005
4	HALLMARK_G2M_CHECKPOINT	197	-0.35	-1.51	0.002	0.024

b. GSEA 'Hallmark gene sets' depleted in MLS 402-91 upon *YAP1* knockdown.

	Gene set	Gene set size	ES	NES	NOM p-value	FDR q-value
1	HALLMARK_G2M_CHECKPOINT	197	-0.56	-2.68	0.000	0.000
2	HALLMARK_MYC_TARGETS_V2	57	-0.67	-2.64	0.000	0.000
3	HALLMARK_MYC_TARGETS_V1	199	-0.53	-2.56	0.000	0.000
4	HALLMARK_E2F_TARGETS	200	-0.53	-2.52	0.000	0.000
5	HALLMARK_CHOLESTEROL_HOMEOSTASIS	70	-0.55	-2.20	0.000	0.000
6	HALLMARK_MITOTIC_SPINDLE	196	-0.41	-1.97	0.000	0.001
7	HALLMARK_MTORC1_SIGNALING	193	-0.42	-1.96	0.000	0.001
8	HALLMARK_SPERMATOGENESIS	73	-0.42	-1.71	0.003	0.004
9	HALLMARK_UNFOLDED_PROTEIN_RESPONSE	111	-0.36	-1.59	0.000	0.012
10	HALLMARK_ANDROGEN_RESPONSE	86	-0.37	-1.52	0.014	0.021
11	HALLMARK_FATTY_ACID_METABOLISM	127	-0,32	-1.40	0,008	0,045
12	HALLMARK_OXIDATIVE_PHOSPHORYLATION	198	-0,29	-1.35	0,010	0,063

c. GSEA 'Hallmark gene sets' enriched in MLS 402-91 upon *FUS-DDIT3* knockdown.

	Gene set	Gene set size	ES	NES	NOM p-value	FDR q-value
1	HALLMARK_ESTROGEN_RESPONSE_EARLY	162	0.51	2.16	0.000	0.000
2	HALLMARK_XENOBIOTIC_METABOLISM	138	0.52	2.13	0.000	0.000
3	HALLMARK_ESTROGEN_RESPONSE_LATE	158	0.50	2.13	0.000	0.000
4	HALLMARK_INFLAMMATORY_RESPONSE	125	0.51	2.08	0.000	0.000
5	HALLMARK_IL6_JAK_STAT3_SIGNALING	60	0.56	2.01	0.000	0.001
6	HALLMARK_APICAL_SURFACE	35	0.53	1.72	0.006	0.006
7	HALLMARK_TNFA_SIGNALING_VIA_NFKB	178	0.40	1.71	0.000	0.006
8	HALLMARK_ANGIOGENESIS	29	0.54	1.65	0.015	0.010
9	HALLMARK_KRAS_SIGNALING_UP	127	0.40	1.63	0.000	0.012
10	HALLMARK_HEME_METABOLISM	166	0.38	1.63	0.000	0.011
11	HALLMARK_COMPLEMENT	145	0.39	1.61	0.000	0.012
12	HALLMARK_BILE_ACID_METABOLISM	72	0.44	1.61	0.004	0.012
13	HALLMARK_ALLOGRAFT_REJECTION	112	0.40	1.57	0.000	0.016
14	HALLMARK_APOPTOSIS	136	0.39	1.57	0.004	0.016
15	HALLMARK_P53_PATHWAY	181	0.36	1.55	0.000	0.017
16	HALLMARK_IL2_STAT5_SIGNALING	149	0,38	1,55	0,006	0,016
17	HALLMARK_EPITHELIAL_MESENCHYMAL_TRANSITION	179	0,35	1,50	0,006	0,026
18	HALLMARK_ADIPOGENESIS	181	0,34	1,48	0,006	0,030
19	HALLMARK_HYPOXIA	168	0,34	1,45	0,004	0,036
20	HALLMARK_FATTY_ACID_METABOLISM	127	0,35	1,42	0,022	0,045
21	HALLMARK_MYOGENESIS	143	0,34	1,40	0,008	0,050
22	HALLMARK_INTERFERON_GAMMA_RESPONSE	161	0,33	1,37	0,016	0,060
23	HALLMARK_UV_RESPONSE_UP	141	0,33	1,35	0,028	0,069
24	HALLMARK_MTORC1_SIGNALING	193	0,31	1,34	0,013	0,068

d. GSEA 'Hallmark gene sets' enriched in MLS 402-91 upon *YAP1* knockdown.

	Gene set	Gene set size	ES	NES	NOM p-value	FDR q-value
1	HALLMARK_P53_PATHWAY	181	0.63	2.56	0.000	0.000
2	HALLMARK_INTERFERON_ALPHA_RESPONSE	90	0.63	2.33	0.000	0.000
3	HALLMARK_INTERFERON_GAMMA_RESPONSE	161	0.54	2.19	0.000	0.000
4	HALLMARK_TNFA_SIGNALING_VIA_NFKB	178	0.41	1.67	0.000	0.012
5	HALLMARK_APOPTOSIS	136	0.40	1.61	0.003	0.018
6	HALLMARK_ALLOGRAFT_REJECTION	112	0.41	1.60	0.003	0.019
7	HALLMARK_KRAS_SIGNALING_DN	90	0.42	1.56	0.010	0.024
8	HALLMARK_IL6_JAK_STAT3_SIGNALING	60	0.45	1.56	0.012	0.021
9	HALLMARK_INFLAMMATORY_RESPONSE	125	0.39	1.55	0.001	0.021
10	HALLMARK_KRAS_SIGNALING_UP	127	0.39	1.53	0.008	0.025
11	HALLMARK_MYOGENESIS	143	0.37	1.46	0.009	0.048
12	HALLMARK_COAGULATION	89	0.39	1.44	0.032	0.052
13	HALLMARK_HEME_METABOLISM	166	0.34	1.39	0.012	0.080
14	HALLMARK_PANCREAS_BETA_CELLS	15	0.51	1.34	0.145	0.116
15	HALLMARK_ESTROGEN_RESPONSE_EARLY	162	0.33	1.34	0.040	0.110

Supplementary Table S3. Antibodies used for immunoblotting (IB), immunohistochemistry (IHC), co-immunoprecipitation (Co-IP) experiments and proximity ligation assays (PLAs).

					Dilution			
**Antibody**	**Clone**	**Company**	**Catalog #**	**Species**	**IB**	**IHC**	**Co-IP**	**PLA**
AKT	-	Cell Signaling Technology	9272	Rabbit	1:1000	-	-	-
β-actin	AC-15	Sigma-Aldrich	A5441	Mouse	1:10.000	-	-	-
CEBP Beta	E299	abcam	ab32358	Rabbit	1:1000	-	-	1:200
CHOP/DDIT3	L63F7	Cell Signaling Technology	2895	Rabbit	1:1000	-	1:80	1:400 - 1:700
FOXM1 XP	D12D5	Cell Signaling Technology	5436	Rabbit	1:1000	-	-	-
GAPDH XP	D16H11	Cell Signaling Technology	5174	Rabbit	1:1000	-	-	-
Histone H3 XP	D1H2	Cell Signaling Technology	4499	Rabbit	1:2000	-	-	-
IGF-I Receptor β XP	D23H3	Cell Signaling Technology	9750	Rabbit	1:1000	-	-	-
IGF-II	S1F2	Merck Millipore	05-166	Mouse	-	1:50	-	-
IGF-I Receptor β	-	Cell Signaling Technology	3027	Rabbit	-	1:100	-	-
LATS1	C66B5	Cell Signaling Technology	3477	Rabbit	1:1000	-	-	-
MOB1	E1N9D	Cell Signaling Technology	13730	Rabbit	1:1000	-	-	-
Mouse IgG2a Isotype Control	E5Y6Q	Cell Signaling Technology	61656	-	-	-	assay dependent	-
Normal Rabbit IgG	-	Cell Signaling Technology	2729	-	-	-	assay dependent	-
Perilipin-1 XP	D1D8	Cell Signaling Technology	9349	Rabbit	1:800	-	-	-
Phospho-AKT (Ser473)	-	Cell Signaling Technology	9271	Rabbit	1:1000	-	-	-
Phospho-IGF-IR β (Tyr1135/1136)	19H7	Cell Signaling Technology	3024	Rabbit	1:1000	-	-	-
Phospho-LATS1 (Thr1079)	D57D3	Cell Signaling Technology	8654	Rabbit	1:1000	-	-	-
Phospho-MOB1 (Thr35)	D2F10	Cell Signaling Technology	8699	Rabbit	1:1000	-	-	-
Phospho-YAP (Ser127)	D9W2I	Cell Signaling Technology	13008	Rabbit	1:1000	-	-	-
Phospho-YAP (Ser397)	D1E7Y	Cell Signaling Technology	13619	Rabbit	1:1000	-	-	-
PI3 Kinase p110α	C73F8	Cell Signaling Technology	4249	Rabbit	1:1000	-	-	-
PPARγ	C26H12	Cell Signaling Technology	2435	Rabbit	1:750	-	-	-
Survivin	71G4B7	Cell Signaling Technology	2808	Rabbit	1:1000	-	-	-
TEAD1/TEF-1	H-4	Santa Cruz	sc-376113	Mouse	1:1000	-	-	1:100
TEAD4/TEF-1	EPR15629]	abcam	ab197589	Rabbit	1:1000	-	-	-
YAP XP	D8H1X	Cell Signaling Technology	14074	Rabbit	1:1000	1:100	1:50	1:100
YAP/TAZ	D24E4	Cell Signaling Technology	8418	Rabbit	1:1000	-	-	-

Supplementary Table S4. List of primers used for quantitative reverse transcription PCR (RT-qPCR).

Target gene (human)	Forward (5'-3')	Reverse (5'-3')
ABCA1	ACCCACCCTATGAACAACATGA	GAGTCGGGTAACGGAAACAGG
ACO2	CCCTACAGCCTACTGGTGACT	TGTACTCGTTGGGCTCAAAGT
ACTB	GACCTGTACGCCAACACAGT	CTCAGGAGGAGCAATGATCT
ADIPOQ	AACATGCCCATTCGCTTTACC	TAGGCAAAGTAGTACAGCCCA
Adipsin	AGGGTCACCCAAGCAACAAAG	TACGTGGCCCATGCTGATCT
APLP2	TGAGCCTCAAATCGCAATGTT	CCTGTTGGATCAGGTTCCCAT
BCL2L11	TAAGTTCTGAGTGTGACCGAGA	GCTCTGTCTGTAGGGAGGTAGG
BCL2L13	AGGACTATTCGGCAGAGTACAT	TGATTCCAGGGTATTCCTCCTC
BIK	GACCTGGACCCTATGGAGGAC	CCTCAGTCTGGTCGTAGATGA
BLCAP	GTGAATAATGGCACTGACCG	AAGGGAAACCAGAAGAGGGA
C/EBP α	TGGACAAGAACAGCAACGAGTA	ATTGTCACTGGTCAGCTCCAG
CBX1	GGTGGAAAAAGTTCTCGACCG	CCCATGTGTTGTCCTCATCTG
CBX3	TAGATCGACGTGTAGTGAATGGG	TGTCTGTGGCACCAATTATTCTT
CCNE1	GCCAGCCTTGGGACAATAATG	CTTGCACGTTGAGTTTGGGT
CDKN1B	ATCACAAACCCCTAGAGGGCA	GGGTCTGTAGTAGAACTCGGG
CMPK1	GGAAGGCAGATGTATCTTTCGTT	TGTTGACTGAAGGTAGGTCTGA
DHRS7	AGCTTAACTACTTAGGGACGGT	CCCAGGATGCTATTCACAGTAAC
E2F4	CACCACCAAGTTCGTGTCCC	GCGTACAGCTAGGGTGTCA
ENPP2	ACTTTTGCCGTTGGAGTCAAT	GGAGTCTGATAGCACTGTAGGA
ETF1	ATACAGAGGCTCTTACAGCACT	AATTTGTGCAGGACTTCTCTTGT
FABP4	ACTGGGCCAGGAATTTGACG	CTCGTGGAAGTGACGCCTT
FAH	GGAGAATGCGTTGATGCCAAA	CATATACGGGAGGCTTAGAGTCA
FUS-DDIT3 (ex7-2)	AGTGGTGGCTATGAACCCAG	AAAGGCAATGACTCAGCTGC
G3BP2	GTTGTGGAACTTCGCATCAATAC	CCCCTCGAAACATAATCGGTTTT
GAPDH	CTCTGCTCCTCCTGTTCGAC	TTAAAAGCAGCCCTGGTGAC
HDDC2	GGGCAGCTCAAGAGAGTCC	GGCGTACACATCGGTCTTTGT
HSPB8	CTCCTGCCACTACCCAAGC	GGCCAAGAGGCTGTCAAGT
LGALS3	ATGGCAGACAATTTTTCGCTCC	GCCTGTCCAGGATAAGCCC
LMNB1	ACATGGAAATCAGTGCTTACAGG	GGGATACTGTCACACGGGA
MCM6	ACCTGCCTACCAGACACAAGA	ACAGAAAAGTTCCGCTCACAAG
PLIN1	TGGGTGGTGTGGCACATAC	CCTCCCCTTGGTTGAGGAGA
PNN	CCAGAAGAGAATCACGCCAGG	GCCGGTTCCTTTGCTTTCC
PPAR γ*2*	GGTGAAACTCTGGGAGATTCT	CTCTGTGTCAACCATGGTCA
PRC1	ATCACCTTCGGGAAATATGGGA	TCTTTCTGACAGACGGATATGCT
RETSAT	TACTTGGGACTATTCTCTGGCA	GCACTTGGTTGGCTGAAAAAG
RIOK3	AATATGATGCACAGCTTAGGCG	ATCACGAGTATCCTGCCAGTC
RNF11	CTGCTTCACGAGTCTCAGTCC	TCTTCCAGGGTCATAAACTCCTT
SNCG	TGAGCAGCGTCAACACTGTG	GAGGTGACCGCGATGTTCTC
STOM	CACACACGGGACTCCGAAG	ATGAGAACGCCACCAAAATCC
TAP1	CTGGGGAAGTCACCCTACC	CAGAGGCTCCCGAGTTTGTG
TGFBR3	GTGTTCCCTCCAAAGTGCAAC	AGCTCGATGATGTGTACTTCCT
TKT	TCCACACCATGCGCTACAAG	CAAGTCGGAGCTGATCTTCCT
TNFSF9	GGCTGGAGTCTACTATGTCTTCT	ACCTCGGTGAAGGGAGTCC
TXNIP	GGTCTTTAACGACCCTGAAAAGG	ACACGAGTAACTTCACACACCT
UBE2C	TGATGTCTGGCGATAAAGGGA	AGCGAGAGCTTATACCTCAGG
VAMP8	TGTGCGGAACCTGCAAAGT	CTTCTGCGATGTCGTCTTGAA
YAP1	GCAAATTCTCCAAAATGTCAGG	CGGGAGAAGACACTGGATTT

Supplementary Table S5: STAR alignment parameters (version 2.5.3a).

Parameter	Value
--alignIntronMax	1100000
--alignIntronMin	20
--alignMatesGapMax	1100000
--alignSJstitchMismatchNMax	5 -1 5 5
--alignSJDBoverhangMin	3
--chimJunctionOverhangMin	15
--chimScoreMin	1
--chimScoreJunctionNonGTAG	0
--chimSegmentMin	15
--chimSegmentReadGapMax	3
--clip3pAdapterSeq	AGATCGGAAGAGCACACGTCTGAACTCCAGTCA
--genomeLoad	NoSharedMemory
--limitBAMsortRAM	1E+11
--outBAMsortingThreadN	1
--outSAMstrandField	intronMotif
--outSAMtype	BAM Unsorted SortedByCoordinate
--outSAMunmapped	Within KeepPairs
--outFilterMismatchNmax	5
--outFilterMismatchNoverLmax	0.3
--outFilterMultimapNmax	1
--readFilesCommand	gunzip -c
--runThreadN	8
--sjdbOverhang	200
--twopass1readsN	-1
--twopassMode	Basic
